# Genomic Evolution and Nitrogen Response Analysis of *Glutamate Synthase* Gene Family in Rice Source–Sink Tissues During Grain Filling

**DOI:** 10.3390/genes17070791

**Published:** 2026-07-12

**Authors:** Shuai Fu, Zixin Xiang, Yuelin Wu, Huihui Zhang, Haiting Hu, Zhuocheng Liu, Han Yang

**Affiliations:** 1 Anhui Province Key Laboratory of Plant Resources and Biology, Anhui Engineering Research Center for Green Production Technology of Drought Grain Crops, College of Life Sciences, Huaibei Normal University, Huaibei 235000, China; 17356152707@163.com (S.F.); 19733887713@163.com (Z.X.); 17756987437@163.com (Y.W.); 19733889023@163.com (H.Z.); hht112119@163.com (H.H.); 2 School of Biological and Food Engineering, Suzhou University, Suzhou 234000, China

**Keywords:** *Oryza sativa*, *glutamate synthase* (*GOGAT*), expression profiling, haplotype diversity, nitrogen stress, enzyme activity

## Abstract

Background/Objectives: Rice (*Oryza sativa*) is the staple food for over half the global population, and nitrogen availability is the primary limiting factor determining rice yield. As the rate-limiting enzyme in nitrogen assimilation and allocation, *glutamate synthase* (*GOGAT*) plays an irreplaceable role throughout the plant life cycle. The evolutionary history, natural genetic variation, and regulatory networks of the *GOGAT* family in rice source–sink tissues during grain filling remain largely elusive. Methods: Here, we combined comparative genomics, population genetics, transcriptomic and biochemical approaches to systematically characterize the *GOGAT* gene family. Genome-wide identification was performed across 12 angiosperm species, followed by haplotype analysis using resequencing data from ~2000 rice accessions. Transcriptomic, enzymatic activity and metabolite content determination were integrated to investigate their responses to three nitrogen gradient treatments in source (roots, flag leaves) and sink (developing embryos) tissues. Results: A total of 48 *GOGAT* genes were identified, clustered into two ancient subfamilies (*GLU*/*GLT*), with a Poaceae-specific duplication event generating *GLT1* and *GLT2* subgroups. Specifically, three rice *GOGAT* genes exhibited distinct domestication signatures: *Fd-GOGAT* showed strong indica-japonica subspecific differentiation, while *NADH-GOGAT2* harbored tropical japonica-specific haplotypes. Furthermore, tissue-specific and developmental stage-dependent nitrogen response patterns were revealed, identifying 5 days after pollination as the critical metabolic switch point. *OsGOGAT* promoters are enriched with light-, ABA- and stress-responsive cis-elements, suggesting coordinated hormonal and environmental regulation. Conclusions: This study provides comprehensive insights into the functional divergence of the plant *GOGAT* gene family and coordinated strategies that rice employs under exogenous nitrogen stress, and identifies elite haplotypes for nitrogen-efficient rice breeding.

## 1. Introduction

Nitrogen (N) is the most essential macronutrient required for plant growth and development, serving as a core structural component of proteins, nucleic acids, chlorophyll, hormones and numerous secondary metabolites [1,2,3]. In agricultural ecosystems, nitrogen availability is universally recognized as the primary limiting factor determining crop yield and grain quality [4]. Rice (*Oryza sativa*) is the staple food for more than half of the global population, and its sustainable production is directly linked to global food security and social stability [5]. To meet the ever-increasing food demand driven by population growth, farmers have relied heavily on synthetic nitrogen fertilizers over the past six decades [6,7,8]. However, excessive nitrogen application has caused a series of severe environmental problems, including soil acidification, groundwater contamination, freshwater eutrophication and increased greenhouse gas emissions [9,10,11]. Moreover, the low nitrogen use efficiency (NUE) of modern rice varieties—typically less than 40%—means that more than 60% of applied nitrogen is lost to the environment, resulting in substantial economic waste and ecological damage [12,13,14]. Therefore, improving crop NUE through molecular breeding has become an urgent priority for sustainable agricultural development worldwide [15,16,17,18,19].

The glutamine synthetase/glutamate synthase (GS/GOGAT) cycle constitutes the central and only pathway for inorganic nitrogen assimilation in higher plants [20]. In this cycle, GS catalyzes the ATP-dependent conversion of glutamate and ammonium to glutamine, while GOGAT subsequently transfers the amide group from glutamine to 2-oxoglutarate to produce two molecules of glutamate. As the rate-limiting enzyme in this cycle, GOGAT plays an irreplaceable role in regulating nitrogen assimilation, metabolism and allocation throughout the plant life cycle [12,21,22,23,24]. Plant GOGAT enzymes are classified into two distinct types based on their electron donors: ferredoxin-dependent GOGAT (Fd-GOGAT) and NADH-dependent GOGAT (NADH-GOGAT). Fd-GOGAT is primarily localized in chloroplasts of photosynthetic tissues, where it is responsible for assimilating ammonium released from photorespiration and nitrate reduction [13,25]. In contrast, NADH-GOGAT is mainly found in non-photosynthetic tissues such as roots and developing seeds, where it participates in primary nitrogen assimilation from soil and nitrogen remobilization from senescing organs to reproductive sinks [26,27,28,29]. Previous studies in *Arabidopsis thaliana* have demonstrated that Fd-GOGAT is essential for photorespiratory nitrogen recycling, and its knockout leads to severe growth retardation under ambient CO_2_ conditions [30,31,32]. In maize (*Zea mays*), NADH-GOGAT has been shown to be specifically expressed in developing kernels and is closely associated with grain protein content and yield [26,33,34].

Despite significant progress in understanding *GOGAT* functions in model plants, the evolutionary history and natural genetic variation in the *GOGAT* gene family in rice remain poorly characterized. Most previous studies have focused on the functional analysis of individual *GOGAT* genes in rice, and there is a lack of comprehensive genome-wide comparative analysis of the entire *GOGAT* family across multiple monocot and dicot species [12,35]. Furthermore, while several studies have investigated the expression patterns of *OsGOGAT* genes under different nitrogen conditions, these studies have primarily focused on vegetative growth stages [25,36]. The regulatory mechanisms of GOGAT-mediated nitrogen assimilation during reproductive growth—particularly the coordinated regulation between source tissues (roots and flag leaves) and sink tissues (developing seeds)—remain largely unknown. Developing seeds, especially young embryos at the early grain-filling stage, are the major nitrogen sink tissues during reproductive growth. The development of young embryos is highly sensitive to nitrogen availability, and their nitrogen assimilation capacity directly determines grain number, grain weight and final yield. However, to date, systematic response profiling of *GOGAT* genes in both source and sink tissues at the transcriptional, enzymatic and metabolic levels under different nitrogen regimes during grain filling needs further exploration.

This study defines four interconnected objectives focused on the *GOGAT* gene family, with rice as the core research system. First, it delineates the evolutionary trajectory and subfamily diversification of *GOGAT* across angiosperms, identifying lineage-specific duplication events and genome-wide selection pressures underlying functional divergence between *NADH-dependent* (*GLT*) and *ferredoxin-dependent* (*GLU*) isoforms in monocot and dicot lineages. Second, it characterizes natural haplotype diversity and domestication footprints of three functional rice GOGAT genes using ~2000 cultivated and wild rice accessions, resolving indica-japonica subspecific differentiation and population-specific haplotypes linked to adaptive nitrogen use traits. Third, with developing reproductive sink tissues as the central focus, it compares tissue-specific nitrogen responses of source (roots, flag leaves) and sink (young embryos) organs at transcriptional, enzymatic and metabolic levels under gradient nitrogen regimes (0N, N-deficient; 1N, moderate N; 3N, excessive N), dissecting non-redundant roles of individual OsGOGAT members in nitrogen assimilation and source–sink remobilization during grain filling. Fourth, it pinpoints the critical developmental switch point of nitrogen metabolism in early seed development and elucidates how exogenous nitrogen availability modulates GOGAT-mediated source–sink nitrogen allocation during reproductive growth. Collectively, this work provides systematic insights into *GOGAT* evolutionary and functional diversification, identifies elite haplotypes as markers for nitrogen-efficient rice breeding, and establishes a scientific basis for precision nitrogen management during grain filling [4,6,11].

## 2. Materials and Methods

### 2.1. Genome-Wide Identification of Glutamate Synthase (GOGAT) Gene Family

Genome-wide protein sequences and chromosome-level genome annotation files of 12 phylogenetically representative angiosperm species, including six Poaceae monocots (*O. sativa*, *Triticum aestivum*, *Z. mays*, *Sorghum bicolor*, *Hordeum vulgare*, *Setaria italica*) and six dicotyledonous species (*A. thaliana*, *Chenopodium quinoa*, *Solanum lycopersicum*, *Glycine max*, *Arachis hypogaea*, *Brassica napus*), were downloaded from the NCBI Gene Database (https://www.ncbi.nlm.nih.gov/genome/) (accessed on 10 April 2026) and the Ensembl Plants database (https://plants.ensembl.org/index.html) (accessed on 10 April 2026). Full-length GOGAT protein sequences of *A. thaliana*, *G. max*, and *A. hypogaea* were retrieved from the corresponding authoritative species-specific genome databases: The Arabidopsis Information Resource (TAIR, https://www.arabidopsis.org/) (accessed on 10 April 2026) [37], SoyBase (https://www.soybase.org/) (accessed on 12 April 2026), and PeanutBase (https://www.peanutbase.org/) (accessed on 12 April 2026), respectively.

Genome-wide identification of the *GOGAT* gene family was performed using a dual strategy combining Hidden Markov Model (HMM)-based search (http://augustus.gobics.de) (accessed on 11 April 2026) [38] and BLAST (https://blast.ncbi.nlm.nih.gov/Blast.cgi?PROGRAM=blastp&PAGE_TYPE=BlastSearch&LINK_LOC=blasthome) (accessed on 11 April 2026) [39] sequence alignment. The HMM profile corresponding to the conserved GOGAT domain (PF01645) was obtained from the Pfam protein family database (https://pfam.xfam.org/) (accessed on 13 April 2026). Homology searches against the whole-genome protein sequences of the 12 aforementioned species were conducted using HMMER software (v3.0), with an E-value cutoff of <1 × 10^−4^ to obtain preliminary candidate sequences. After removing redundant and truncated sequences, the remaining preliminary candidates were submitted to the SMART platform (https://smart.embl.de/) (accessed on 13 April 2026) and NCBI Conserved Domain Database (CDD, https://www.ncbi.nlm.nih.gov/cdd/) (accessed on 13 April 2026) for conserved domain validation. Only sequences harboring the intact conserved Glu_synthase domain were retained as the final identified *GOGAT* gene set, with full annotation details provided in Table S1.

Chromosomal localization analysis of the three *OsGOGAT* genes was performed, and their chromosomal distribution map was generated and visualized using TBtools software (v2.0) [40].

### 2.2. Phylogenetic Tree Construction of the GOGAT Gene Family

To elucidate the evolutionary relationships and divergence patterns of the *GOGAT* gene family across diverse plant lineages, multiple sequence alignment of full-length GOGAT protein sequences from the 12 aforementioned plant species was performed using MEGA 11 software (http://www.megasoftware.net/) with default parameters. The resulting high-quality alignment matrix was used as the foundational dataset for phylogenetic inference. Based on this matrix, a maximum likelihood (ML) phylogenetic tree of the *GOGAT* gene family was constructed using FastTree software (http://www.microbesonline.org/fasttree/) (accessed on 12 April 2026). Branch support was assessed via 1000 bootstrap replicates [41] to evaluate the statistical robustness of the inferred clades, with bootstrap values of 70% or higher considered to indicate strong branch support. All the other parameters were set to default. Visualization, annotation and refinement of the final phylogenetic tree were completed using the Interactive Tree of Life (iTOL) platform (https://itol.embl.de/) (accessed on 10 April 2026).

### 2.3. Cross-Species Collinearity Analysis of the GOGAT Gene Family in Oryza sativa

Genome-wide syntenic relationships between *O. sativa* and six representative plant species (*T. aestivum*, *Z. mays*, *S. bicolor*, *A. thaliana*, *G. max*, *and B. napus*) were analyzed using the built-in MCScanX module of TBtools software (v2.0) [42], through which syntenic homologous *GOGAT* gene pairs were identified and quantified. Genome-wide synteny circos plots and *GOGAT* gene-specific syntenic link diagrams were generated using the Basic CIRCOS function of TBtools. In these plots, gray lines represent genome-wide syntenic blocks between species, while red lines highlight the syntenic homologous gene pairs of the *GOGAT* family.

### 2.4. Gene Structure Analysis of the GOGAT Gene Family in Oryza sativa

The positional information of coding sequences (CDSs), 5′ untranslated regions (5′ UTR), 3′ untranslated regions (3′ UTR), exons, and introns for all *OsGOGAT* genes was extracted from the *O. sativa* reference genome annotation file. The exon-intron architecture of *OsGOGAT* genes was visualized using the “Gene Structure View” module of TBtools software (v2.0) [42]. Full-length gene sequences, exon counts, and intron length information were preserved during visualization to accurately depict the genomic structural characteristics of the *OsGOGAT* gene family.

### 2.5. Multiple Sequence Alignment of GOGAT Proteins from Different Plant Species

Multiple sequence alignment of full-length GOGAT protein sequences from six representative plant species, including *O. sativa*, *T. aestivum*, *Z. mays*, *A. thaliana*, *G. max*, and *B. napus*, was performed using CLUSTALW software (v2.1), with all the alignment parameters set to the program default values [43]. Separate independent multiple sequence alignment analyses were conducted for the two functional GOGAT subfamilies, namely ferredoxin-dependent GOGAT (GLU) and NADH-dependent GOGAT (GLT), according to their functional classification. Based on the alignment results, publication-quality standardized sequence alignment maps were generated using the online visualization tool ESPript 3.0 (https://espript.ibcp.fr/ESPript/ESPript/) (accessed on 11 April 2026), with conserved amino acid residues and the corresponding positions of conserved functional domains (GATase_2, Glu_syn_central, Glu_synthase and GXGXG) of GOGAT proteins clearly annotated.

### 2.6. Prediction and Visualization Analysis of Cis-Acting Elements in the Promoter Regions of GOGAT Genes

To explore the putative cis-acting regulatory elements involved in the transcriptional regulation of the *GOGAT* gene family, the 2000 bp genomic sequences upstream of the translation initiation codon (ATG) of each *GOGAT* gene were extracted from the genome annotation files of four representative plant species, including *O. sativa*, *T. aestivum*, *A. thaliana*, and *G. max*, and were defined as the promoter regions for subsequent analysis [44]. Prediction of cis-acting elements within the obtained promoter sequences was performed using the PlantCARE online database (http://bioinformatics.psb.ugent.be/webtools/plantcare/html/) (accessed on 11 April 2026), followed by the screening of statistically significant regulatory elements. Based on the prediction results, visualization analysis of the distribution and abundance characteristics of the identified cis-acting elements was conducted using TBtools software (v2.0).

### 2.7. Conserved Motif Analysis of GOGAT Proteins

Protein domain identification and localization were performed using Pfam-Scan software against the Pfam database. De novo identification and analysis of conserved motifs in GOGAT protein sequences from *O. sativa*, *T. aestivum*, *A. thaliana*, and *G. max* were conducted using the MEME online tool (https://meme-suite.org/meme/tools/meme) (accessed on 13 April 2026) [45]. The analysis was performed in classic mode with the zero-or-one-occurrence-per-sequence (zoops) site distribution model. A maximum of 30 motifs were detected [46], with the optimal motif width constrained to 20–150 amino acids, while all the other parameters were set to default values. Based on the MEME results, the number, type, and distribution patterns of conserved GOGAT motifs were visualized using TBtools software (v2.0). HMM sequence logos were generated for core conserved motifs. Integrated with the phylogenetic clustering results, the distribution patterns of subfamily-specific motifs and their precise correspondence to the functional domains GATase_2, Glu_synthase, GXGXG, and Glu_syn_central were systematically characterized.

### 2.8. Structural Prediction and Functional Analysis of Interaction Networks of GOGAT Proteins in Oryza sativa

Protein–protein interaction (PPI) networks of OsGOGAT proteins were constructed using the STRING database (https://string-db.org/) (accessed on 10 April 2026) [47]. High-confidence interacting proteins were screened and subjected to functional correlation analysis. Gene Ontology (GO) enrichment analysis was performed on the identified interacting proteins to elucidate the key biological processes in which GOGAT proteins participate. Secondary structures of OsGOGAT proteins were predicted using the SOPMA online tool (http://npsa-pbil.ibcp.fr/cgi-bin/npsa_automat.pl?page=npsa_sopma.html) (accessed on 12 April 2026), and the proportional characteristics of α-helices, extended strands, β-turns, and random coils were quantified. Three-dimensional (3D) homology modeling of the three OsGOGAT proteins was conducted using AlphaFold2 (https://alphafold.ebi.ac.uk/) (accessed on 13 April 2026), and structural visualization was performed using PyMOL (v3.0) to generate high-confidence 3D structural models of these proteins. For OsGOGAT proteins, PPI networks were further constructed based on the STRING database with the species specified as *O. sativa*, and high-confidence interacting proteins were filtered [48]. The interaction networks were visualized using Cytoscape (v3.8), and GO functional enrichment analysis of the interacting proteins was implemented using the clusterProfiler R package (v4.6.1), with an adjusted *p*-value < 0.05 defined as the threshold for statistically significant enrichment.

### 2.9. Ka/Ks Analysis of GOGAT Genes

The non-synonymous substitution rate (Ka), synonymous substitution rate (Ks), and Ka/Ks ratio of *GOGAT* orthologous gene pairs between *O. sativa* and six representative angiosperm species (*Z. mays*, *T. aestivum*, *S. bicolor*, *A. thaliana*, *G. max*, and *B. napus*) were systematically determined through an integrated analytical framework with bidirectional cross-validation, combining the BLAST tool hosted on the Gramene-Ensembl Plants platform (https://oryza-ensembl.gramene.org/Oryza_barthii/Tools/Blast?db=core;expand_form=true;tl=GHxeaRhX9LzEoli7-13904#) (accessed on 11 April 2026) and TBtools software (v2.0). In parallel, homologous CDSs of *GOGAT* genes were retrieved from a genetically diverse panel of *Oryza* germplasms, followed by comprehensive multiple sequence alignment (MSA) analysis. A total of 28 Oryza accessions were included in this analysis, comprising 8 wild *Oryza* species (*Oryza punctata*, *Oryza nivara*, *Oryza barthii*, *Oryza meridionalis*, *Oryza glumaepatula*, *Oryza rufipogon*, *Oryza glaberrima*, and *Oryza brachyantha*), 10 cultivars of *O. sativa* spp. *japonica* (CHAO MEO, Azucena, Nipponbare, KETAN NANGKA, Sasanishiki, SAKHA 102, Carolina, ARC 11359, ZhongHua11Hao, and ShenNong265), and 10 cultivars of *O. sativa* spp. *indica* (PR 106, Minghui 63, IR 64, Zhenshan 97, LIMA, KHAO YAI GUANG, GOBOL SAIL, LIU XU, LARHA MUGAD, and N22) in Table S2. Based on the above homologous gene pair sequences, Ka, Ks, and Ka/Ks ratio were calculated using the BLAST tool hosted on the Gramene-Ensembl Plants platform and TBtools. Genes with Ka/Ks ratios < 1, >1, and =1 were classified as undergoing purifying selection, positive selection, and neutral evolution, respectively [49]. The distribution characteristics of Ka/Ks ratios were visualized via boxplots to dissect the evolutionary selection patterns of the *GOGAT* gene family.

### 2.10. Haplotype Analysis of GOGAT Genes in Oryza sativa

The 1500 bp promoter and full-length coding sequence of *NADH-dependent GOGAT1* (*NADH-GOGAT1*), *NADH-GOGAT* and *Fd-GOGAT* in 2005, 1999 and 1992 rice accessions were obtained from the MBKbase rice repository (https://mbkbase.org) (accessed on 11 April 2026). The cultivated panel comprised approximately 2005 _*O. sativa*_ accessions aggregated from nine pre-release projects within MBKbase (HBWild461, XD248, HB950, Japan176, MBK1287, HXH67, Rice3k, ISG575, HAU533). Sub-population assignments (Indica, Basmati/Sadri, Aus/Boro, Temperate Japonica, Tropical Japonica) were obtained from the rice 3K project (RFGB, http://www.rmbreeding.cn/Index/s) (accessed on 11 April 2026) [50] and collapsed into the legend categories Ind, Bas, Aus, Tem-Jap, and Tro-Jap, respectively. The wild rice panel comprised 19 _Oryza rufipogon_ accessions from the HBWild461 project, drawn from the Or-I, Or-II, Or-III and Wild ecotype groups and merged into a single Wild category (Table S3).

The Nipponbare reference genome was adopted as the uniform coordinate reference for all the genomic analyses in this study. Quality filtering of genomic variants was performed independently for the cultivated rice panel and the wild rice panel. For the cultivated panel, variants meeting all the following criteria were retained for downstream analysis: mapping rate ≥ 80%, genomic coverage ≥ 60%, sequencing depth ≥ 12×, alternative allele frequency ≥ 20%, and missing genotype rate ≤ 0.5%. For the wild panel, the filtering thresholds were set as follows: mapping rate ≥ 80%, genomic coverage ≥ 60%, sequencing depth ≥ 3×, alternative allele frequency ≥ 20%, missing genotype rate ≤ 5%, and at least 1 valid sample per variant site. In addition, wild accessions were retained in the final SNP dataset only if they harbored a minimum of 3 non-N genotype calls across the entire SNP set. Nucleotide diversity curves across the target loci were calculated using a sliding-window algorithm with a window size of 2000 bp and a step size of 100 bp. To dissect the evolutionary history of *GOGAT* genes, phylogenetic trees and haplotype networks were constructed based on qualified variants, with an additional round of quality control implemented prior to analysis using the pegas R package. At the site level, only biallelic SNPs—defined as sites with a 1 bp reference allele, a 1 bp alternative allele, and allelic bases restricted to A, C, G, T and—that were annotated to reside in promoter, 5′ UTR, 3′ UTR, or non-synonymous coding sequence regions. At the sample level, sequences with a missing genotype rate exceeding 80% across the union of all the filtered sites were excluded. Ultimately, all the polymorphic sites within the *GOGAT* locus with a minor allele frequency ≥ 0.005 were used for phylogenetic tree reconstruction and haplotype network construction. The phylogenetic trees were constructed using the UPGMA method by Mega7.0. The haplotype network was generated via the pegas 1.4-x package in the R 4.5.3 statistical environment, with the ape package for phylogenetic data manipulation and svglite for vector graphic export. Data preprocessing and result visualization were carried out in Python 3 using the pandas and matplotlib libraries [51,52].

### 2.11. Plant Materials and Hydroponic Treatments

Seeds of the indica rice cultivar Zhongxian 3037 were used as the experimental plant material. Seedlings were grown hydroponically in an artificial climate incubator maintained at 23–35 °C with a 10 h light/14 h dark photoperiod, 70% relative humidity, and a light intensity of 200 μmol·m^−2^·s^−1^. Plants were cultivated in porous ceramic granules (PROFILE Products LLC, Buffalo Grove, IL, USA) and supplied with standard Kimura B nutrient solution, which was defined as the baseline normal nitrogen (N) condition. The nutrient solution was completely refreshed every 3 days throughout the growth period.

After 50 days of vegetative growth in normal Kimura B nutrient solution, whole rice plants were subjected to three nitrogen nutritional regimes (0N, 1N and 3N). For N concentration gradient treatments, the standard Kimura B formulation was modified as follows: (NH_4_)_2_SO_4_ and KNO_3_ were replaced with NH_4_NO_3_ as the sole N source, and Ca(NO_3_)_2_ was substituted with CaCl_2_·2H_2_O to maintain calcium availability. Three NH_4_NO_3_ concentrations were established for the gradient experiment: 0 mM, 0.9 mM, and 2.7 mM. Relative to the native total N concentration of unmodified Kimura B solution (1.7 mM), the 0 mM NH_4_NO_3_ treatment was designated as the 0N (N-deficient) condition, the 0.9 mM treatment as the 1N (moderate/optimal N) condition, and the 2.7 mM treatment as the 3N (high/excessive N) condition, respectively.

### 2.12. Sample Collection and RNA Isolation

Flag Leaf and Root Tip Collection and Total RNA Extraction: Apical 5 cm segments of flag leaves and 5 cm root tips were harvested from wild-type (WT) Zhongxian 3037 plants subjected to the three nitrogen treatments described above. All the samples were immediately snap-frozen in pre-chilled liquid nitrogen and stored at −80 °C until further processing. Total RNA was isolated using TRIZOL reagent according to the manufacturer’s standard protocols. RNA sequencing (RNA-seq) library construction and high-throughput sequencing were performed by Metware Biotechnology Co., Ltd. (Wuhan, China; https://www.metware.cn/) (accessed on 20 October 2025) (Table S4).

Rice Young Embryo Collection and Total RNA Extraction: Total RNA was extracted from rice embryos collected at four distinct developmental stages from WT plants grown under the three nitrogen gradients. The developmental stages were defined as follows: Stage 1 (1 day after pollination, DAP): zygote polarization stage, characterized by active cell division and no organ differentiation; Stage 2 (2 DAP): globular proembryo stage, with established axial polarity and rapid cell proliferation; Stage 3 (3–4 DAP): organ primordia initiation stage, marked by tissue differentiation and functional transition; Stage 4 (5–6 DAP): mature embryo stage, with fully formed embryonic organs, active nutrient storage and nitrogen metabolism, and rapid accumulation of storage compounds. Following manual dissection under a stereomicroscope, young embryos were immediately snap-frozen in liquid nitrogen. Total RNA was extracted using a magnetic bead-based high-efficiency total RNA extraction kit specifically designed for polysaccharide- and polyphenol-rich plants (TIANGEN Biotech, Cat. No. DP772) following the manufacturer’s instructions.

To ensure the strict uniformity of the samples, samples were loaded into round-bottom centrifugal tubes within steel balls for grinding at a rate of 23 times per second for 3 min after snap-frozen in pre-chilled liquid nitrogen. The size of the centrifugal tubes can be adjusted according to the volume of the sample. During the grinding process, each minute of grinding was followed by 1 min of snap-freezing to prevent the samples from generating friction heat. After tissue homogenization, RNA profiling was conducted. RNA-seq analysis was performed by Metware Biotechnology Co., Ltd. (Wuhan, China; https://www.metware.cn/) (accessed on 20 January 2026) (Table S5). The Nipponbare reference genome was used as the coordinate reference. The expression profiling of *GOGAT* genes was visualized in a bar chart, which was constructed via the Metware Cloud platform (https://cloud.metware.cn/#/tools/tool-list) (accessed on 20 January 2026).

### 2.13. GOGAT Enzyme Activity Determination

The apical 10 cm of flag leaves, the apical 5 cm of root tips, and developing embryos at three developmental stages (1, 5 and 10 DAP) were collected. All the samples were immediately frozen in liquid nitrogen and stored at −80 °C in an ultra-low temperature freezer until further analysis. All the GOGAT enzyme activity assays were performed by the Technical Service Department of Beijing Solarbio Science & Technology Co., Ltd. (Beijing, China; https://solarbio.bioon.com.cn/) (accessed on 25 October 2025) via the GOGAT Activity Detection Kit BC0075 (Table S6).

### 2.14. Glutamic Acid (Glu) Content Determination

Developing embryos at three developmental stages (1, 5 and 10 DAP) were collected and immediately frozen in liquid nitrogen and stored at −80 °C in an ultra-low temperature freezer until further analysis. Glu content determination was performed by the Technical Service Department of Beijing Solarbio Science & Technology Co., Ltd. (https://solarbio.bioon.com.cn/) (accessed on 25 October 2025) (Table S7).

### 2.15. Statistical Analysis

All experiments were performed with three independent biological replicates. Statistical analyses of the data were conducted using GraphPad Prism version 10.1.2 software. Differences between treatment groups were assessed using one-way analysis of variance (ANOVA) followed by Tukey’s honestly significant difference (HSD) post hoc test. The statistical significance threshold was set at *p* < 0.05. For letter-based significance notation: within each individual gene, values from different nitrogen treatment groups sharing the same lowercase letter are not significantly different (*p* > 0.05), whereas values marked with distinct letters indicate statistically significant differences (*p* < 0.05). All the pairwise comparisons were restricted exclusively to treatment groups within the same gene, and no cross-gene statistical comparisons were conducted.

## 3. Results

### 3.1. Identification and Analysis of the Glutamate Synthase (GOGAT) Gene Family

Genome-wide identification of *GOGAT* gene family members across 12 plant species was performed using a Hidden Markov Model (HMM)-based approach. This rigorous filtering pipeline yielded a total of 48 non-redundant GOGAT protein sequences. Copy numbers per species were as follows: 3 in *O. sativa*, 6 in *T. aestivum*, 4 in *Z. mays*, 3 in *S. bicolor*, 2 in *H. vulgare*, 3 in *S. italica*, 3 in *A. thaliana*, 3 in *C. quinoa*, 2 in *S. lycopersicum*, 6 in *G. max*, 6 in *A. hypogaea* and 7 in *B. napus*. Full details of the identified *GOGAT* genes are provided in Table S1.

The chromosomal distribution of genes is tightly correlated with their transcriptional engagement levels and functional significance in plant growth and developmental processes. To elucidate the replication patterns and evolutionary mechanisms of the *OsGOGAT* gene family, chromosomal localization analysis of the three *OsGOGAT* genes was performed using TBtools software, and their genomic distribution profiles were visualized (Figure S1). The three *OsGOGAT* genes were found to be discretely distributed across three distinct *O. sativa* chromosomes: *OsGLT1* (*Os01g0681900*) was mapped to chromosome 1, *OsGLT2* (*Os05g0555600*) to chromosome 5, and *OsGLU1* (*Os07g0658400*) to chromosome 7. No tandemly duplicated gene clusters or segmental duplication events were detected among the *OsGOGAT* family members.

### 3.2. GOGAT Phylogenetic Analyses Across 12 Plant Species

**Figure 1:**
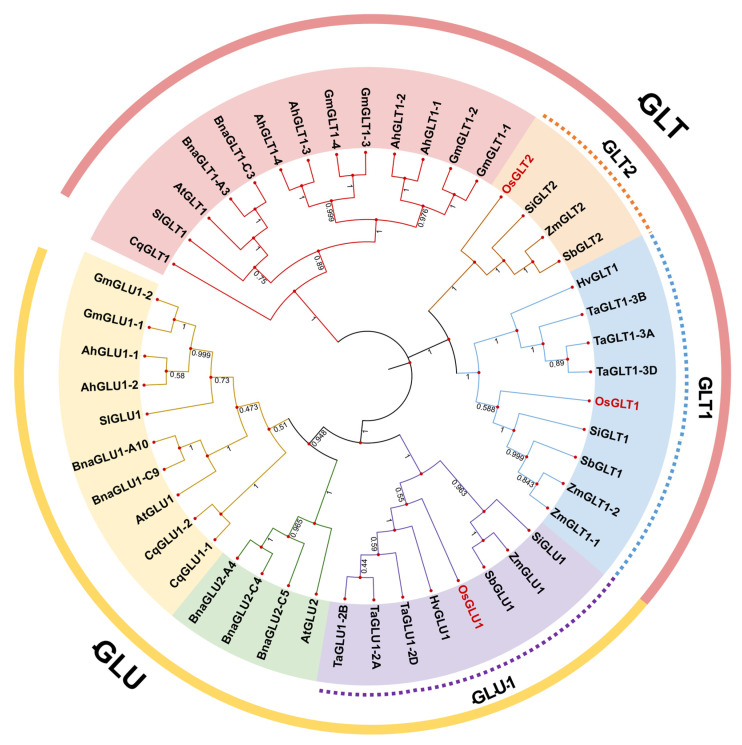
Phylogenetic analysis of GOGAT family proteins in multiple plant species including *Oryza sativa*, *Triticum aestivum*, *Zea mays*, *Sorghum bicolor*, *Hordeum vulgare*, *Setaria italica*, *Arabidopsis thaliana*, *Chenopodium quinoa*, *Solanum lycopersicum*, *Glycine max*, *Arachis hypogaea*, and *Brassica napus*. The tree was divided into two major subfamilies, namely GLT and GLU, and the GLT clade was further classified into two subgroups (GLT1 and GLT2) in Poaceae species according to the OsGOGAT nomenclature. Different colored regions represent distinct subfamily or subgroup classifications, and bootstrap support values are indicated at each node.

To elucidate the evolutionary relationships of the *GOGAT* gene family, phylogenetic analysis was performed using the full-length amino acid sequences of 48 non-redundant GOGAT proteins identified from 12 representative plant species. The resulting tree was visualized using the iTOL platform (Figure 1).

All GOGAT family proteins were clearly clustered into two evolutionarily distinct major subfamilies, corresponding to NADH-dependent GOGAT (GLT) and ferredoxin-dependent GOGAT (GLU). Strikingly, the two subfamilies exhibited reciprocal divergence patterns between monocot and dicot lineages: the GLT subfamily in monocotyledonous plants was further resolved into two well-supported monophyletic subclades designated GLT1 and GLT2, whereas only the GLT1 subclade was detected in dicotyledonous plants. Conversely, the GLU subfamily diversified into GLU1 and GLU2 subclades in dicots but retained only the GLU1 subclade in monocots. This lineage-specific subclade differentiation reflects adaptive evolution of the GOGAT family to meet the distinct nitrogen assimilation requirements of monocot and dicot plants following their evolutionary divergence.

Differential subclade expansion and evolution have occurred across the two functional subfamilies. Analysis of species-specific copy number profiles reveals pronounced, lineage-specific expansions in both the GLU and GLT subfamilies among polyploid species including *T. aestivum*, *B. napus*, *A. hypogaea*, and *G. max*. These findings strongly indicate that whole-genome duplication and segmental duplication events represent the primary evolutionary forces driving copy number expansion of this gene family in polyploid lineages. In contrast, the GOGAT family has evolved in a markedly more conserved manner in diploid species such as *O. sativa* and *S. bicolor*, with each species maintaining a stable complement of exactly three family members.

### 3.3. Collinearity Analysis of GOGAT Genes Between Oryza sativa and Six Representative Plant Species

**Figure 2:**
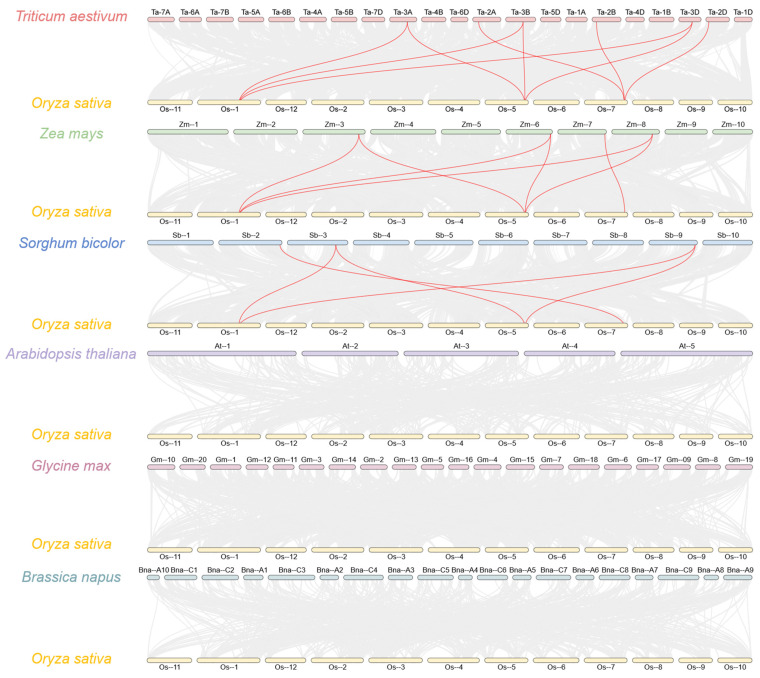
Collinearity analysis of *GOGAT* genes among *Oryza sativa* and other plant species. Collinearity analysis was performed to identify the syntenic relationships of *GOGAT* genes between *O. sativa* and six other species, including *Triticum aestivum*, *Zea mays*, *Sorghum bicolor*, *Arabidopsis thaliana*, *Glycine max*, and *Brassica napus*. Gray lines represent the genome-wide collinear blocks between *O. sativa* and the other species, while red lines highlight the collinear *GOGAT* gene pairs.

To further dissect the evolutionary origin and expansion mechanisms of the *GOGAT* gene family, genome-wide synteny analysis was performed between *O. sativa* and six representative plant species using the MCScanX module in TBtools software (Figure 2).

Syntenic orthologous relationships of *GOGAT* family genes were exclusively detected between *O. sativa* and three Poaceae species (*T. aestivum*, *Z. mays*, *S. bicolor*), with no collinear *GOGAT* gene pairs identified between *O. sativa* and any of the three dicot species (*A. thaliana*, *G. max*, *B. napus*). Specifically, 9, 7, and 5 syntenic *GOGAT* ortholog pairs were identified between *O. sativa* and *T. aestivum*, *Z. mays*, and *S. bicolor*, respectively. Within the Poaceae family, the highest number of syntenic *GOGAT* gene pairs was observed between *O. sativa* and *T. aestivum*, which is highly consistent with the genomic characteristic of *T. aestivum* as an allohexaploid that has undergone multiple whole-genome duplication events, leading to subgenome-level copy number expansion of the *GOGAT* family. The number of syntenic *GOGAT* gene pairs between *O. sativa* and the diploid species *Z. mays* and *S. bicolor* matches their conserved *GOGAT* family copy number profiles, indicating exceptionally high genomic synteny conservation of the *GOGAT* family across Poaceae species. This family has retained intact homologous gene blocks during Poaceae diversification, and its core catalytic function in nitrogen assimilation has been subjected to strong purifying selection constraints throughout evolution.

In stark contrast, the complete absence of syntenic *GOGAT* ortholog pairs between *O. sativa* and all three dicot species reflects extensive chromosomal rearrangements, gene fragment loss, and relocation that have occurred over the long evolutionary timescale following the monocot-dicot divergence approximately 140–150 million years ago, ultimately resulting in the complete erasure of genome-wide synteny for the *GOGAT* gene locus.

### 3.4. Gene Structure Analysis of GOGAT Genes in Oryza sativa

The diversity of gene architectures offers essential insights into the evolutionary dynamics of gene families. To further characterize the *OsGOGAT* gene family, comprehensive gene structure analysis was performed. Exon-intron architectures of the three *OsGOGAT* genes were analyzed based on the *O. sativa* reference genome annotation, and the results were visualized using TBtools software (Figure S2). All three *OsGOGAT* genes contain intact 5′-untranslated regions (5′-UTRs), 3′-untranslated regions (3′-UTRs), and coding sequences (CDSs), exhibiting the characteristic multi-exon-intron structure of eukaryotic genes.

The two members of the *GLT* subfamily, *OsGLT1* and *OsGLT2*, share nearly identical gene architectures, each containing exactly 22 exons with highly conserved exon lengths, arrangement orders, and intron insertion sites. This striking structural conservation reflects the strong evolutionary stability and functional constraint of the *GLT* subfamily. In stark contrast, *OsGLU1*, the sole member of the *GLU* subfamily, possesses 33 exons, a significantly larger number than that of the *GLT* subfamily members. Its genomic length is also markedly longer, with distinct exon arrangement patterns and intron length distributions compared to the *GLT* subfamily. This pronounced structural divergence between the *GLT* and *GLU* subfamilies indicates that gene structural variation has accompanied the functional specialization of these two *GOGAT* isoforms during *O. sativa* evolution.

Transmembrane helices and signal peptides of the three OsGOGAT proteins (OsGLT1, OsGLT2, and OsGLU1) were subjected to in silico prediction analysis. All three proteins are predicted to be globular proteins devoid of transmembrane helices and lack canonical N-terminal signal peptides. These findings collectively demonstrate that OsGOGAT proteins are soluble intracellular proteins that neither localize to cellular membrane systems nor are targeted to the classical secretory pathway (Figure S3). Three-dimensional (3D) structural models of OsGOGAT proteins (OsGLT1, OsGLT2, and OsGLU1) were constructed using SWISS-MODEL. All the models exhibited high structural reliability, with their secondary structure elements predominantly consisting of α-helices and random coils, confirming the high evolutionary conservation of the monomeric assembly pattern of OsGOGAT proteins (Figure S4).

### 3.5. Amino Acid Sequence Alignment of GOGAT Proteins Across Three Monocotyledonous and Three Dicotyledonous Species

To systematically elucidate the sequence conservation of the *GOGAT* gene family, amino acid sequence alignments were separately conducted for GLU proteins (Figure S5) and GLT proteins (Figure S6) from *O. sativa*, *T. aestivum*, *Z. mays*, *A. thaliana*, *G. max*, and *B. napus* using CLUSTALW, and the results were visualized with ESPript 3.0.

Sequence alignment revealed exceptionally high conservation within the core functional domains of the GLU subfamily. The GATase_2 domain for glutamine amidotransferase activity, the Glu_synthase domain as the catalytic core of glutamate synthase, and the GXGXG signature motif for cofactor binding were strictly conserved across all GLU proteins. Amino acids at multiple key catalytic sites were identical in monocot and dicot species, reflecting strong evolutionary constraints on the core catalytic function of nitrogen assimilation in this subfamily.

The core catalytic domains of the GLT subfamily showed equivalent conservation to those of the GLT subfamily. In contrast, the C-terminal NADH-binding domains (Fer4_20 and Pyr_redox_2) exhibited distinct subfamily-specific conservation, with key residues fully preserved across all GLT members. These regions constitute the essential sequence foundation for NADH-dependent electron transfer and chloroplast-localized nitrogen assimilation by the GLT subfamily.

### 3.6. Cis-Acting Element Prediction of GOGAT Genes in Oryza sativa, Triticum aestivum, Arabidopsis thaliana and Glycine max

**Figure 3:**
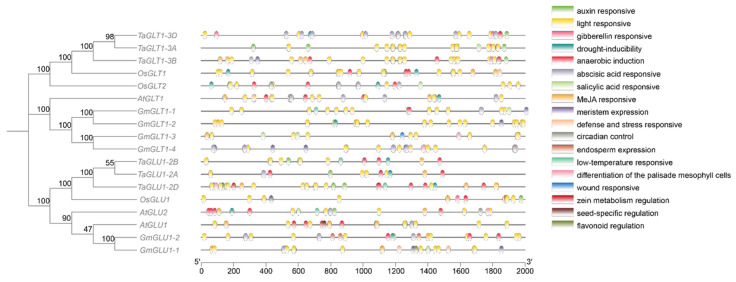
Prediction of cis-acting elements in *GOGAT* genes across diverse plant species. Cis-acting elements in the promoter regions of *GOGAT* genes from *Oryza sativa*, *Triticum aestivum*, *Arabidopsis thaliana*, and *Glycine max* were identified using PlantCARE. The visualization was performed using TBtools, with different colored circles representing distinct cis-elements associated with specific biological functions as indicated in the legend.

In order to deeply investigate the expression regulation mechanism of *OsGOGAT* genes, the cis-acting elements of *OsGOGAT* genes were analyzed in detail. Cis-acting regulatory elements in the promoter regions of *GOGAT* genes from *O. sativa*, *T. aestivum*, *A. thaliana* and *G. max* were predicted using PlantCARE, and the results were visualized with TBtools (Figure 3). Highly conserved functional categories of cis-elements are observed across all four species, with light-responsive elements constituting the predominant class. Abundant light-responsive elements are detected in all *GOGAT* genes, accounting for the highest proportion among all functional element categories, indicating that *GOGAT* expression is universally regulated by light signals. This regulatory pattern is highly consistent with the core biological function of coordinated regulation between photosynthesis and nitrogen assimilation in plants. Furthermore, *GOGAT* genes from all the species are universally enriched in elements responsive to anaerobic induction, abscisic acid (ABA), methyl jasmonate (MeJA), drought, as well as defense and stress responses. These findings demonstrate the evolutionary conservation of *GOGAT* family functions in plant hormone signal transduction and abiotic/biotic stress responses across species, establishing *GOGAT* genes as critical regulatory components for maintaining nitrogen metabolic homeostasis and adapting to environmental stresses in plants.

At the subfamily level, the *GLT* subfamily and *GLU* subfamily exhibit pronounced functional divergence in their cis-element composition. *GLT* subfamily genes are preferentially enriched in light-responsive, ABA-responsive, anaerobic-inducible and drought-inducible elements. Notably, *OsGLT1* and *OsGLT2*, the two *GLT* subfamily members in *O. sativa*, harbor multiple drought- and anaerobic-inducible elements, which are highly congruent with their chloroplastic localization and photosynthesis-coupled nitrogen assimilation function. In contrast, *GLU* subfamily genes are preferentially enriched in MeJA-responsive elements and elements associated with tissue-specific expression, including those regulating meristematic expression, endosperm expression and seed-specific regulation. This cis-element profile aligns perfectly with their roles in nitrogen transport, reassimilation and grain development, which are localized to roots and vascular tissues. Pronounced intraspecific divergence in cis-element composition is also observed among different *GOGAT* members within the same species.

Among the three *OsGOGAT* genes, *OsGLT1* uniquely possesses auxin-responsive elements and defense- and stress-responsive elements; *OsGLT2* exclusively harbors salicylic acid-responsive elements and circadian rhythm regulatory elements. This differential cis-element repertoire indicates functional divergence in environmental responsiveness and expression regulatory patterns between the two *OsGLT* subfamily members. *OsGLU1*, in turn, uniquely contains gibberellin-responsive elements and meristematic expression elements, forming a distinct regulatory signature from that of *GLT* subfamily members.

Furthermore, striking species-specific cis-element divergence is detected across *GOGAT* genes from different species. *TaGOGAT* genes uniquely contain zein metabolism regulatory elements. *GmGOGAT* genes are enriched in elements regulating palisade tissue differentiation, as well as wound- and gibberellin-responsive elements. In Arabidopsis, *AtGLU1* exclusively harbors seed-specific regulatory elements, while *AtGLU2* uniquely possesses low-temperature-responsive and palisade tissue differentiation regulatory elements. These species-specific cis-element profiles reveal that *GOGAT* genes exert differential regulatory roles in species-specific physiological processes across crops, including grain development, storage substance accumulation and environmental stress adaptation.

### 3.7. Conserved Motif Analysis of GOGAT Proteins from Oryza sativa, Triticum aestivum, Arabidopsis thaliana and Glycine max

To characterize the conserved structural features of GOGAT proteins, conserved motif profiling was conducted for *GOGAT* homologs from *O. sativa*, *T. aestivum*, *A. thaliana*, and *G. max* via the MEME tool (Figure S7). A total of 30 conserved motifs were identified across the dataset, with the identity and linear arrangement of these motifs showing a high degree of global conservation among all the analyzed GOGAT proteins. Strikingly, Motif 1 (corresponding to the GATase_2 domain), Motif 2 (corresponding to the Glu_synthase domain), Motif 3 (corresponding to the GXGXG nucleotide-binding motif), and Motif 4 (corresponding to the Glu_syn_central domain) are universally detected in all the GOGAT proteins in a strictly conserved sequential order. These four motifs collectively constitute the core functional framework of glutamate synthases, providing the fundamental sequence basis for the evolutionary stability of their catalytic functions. Subfamily-specific comparative analysis revealed that Motif 16 (corresponding to the Fer4_20 domain) and Motif 17 (corresponding to the Pyr_redox_2 domain) are exclusively restricted to members of the GLT subfamily, representing the defining sequence signatures that discriminate this subfamily from the GLU subfamily (Figure S8).

Proteins within the same GOGAT subfamily display highly consistent motif composition, copy number, and linear arrangement pattern, whereas pronounced divergence in motif distribution is evident between the GLU and GLT subfamilies. This dichotomous pattern is highly congruent with the clustering topology of the phylogenetic tree, which not only reflects the overall evolutionary conservation of the GOGAT family, but also delineates the molecular basis underlying the functional divergence between these two subfamilies.

### 3.8. Prediction of Interacting Proteins and Functional Network Analysis of OsGOGAT Family Proteins

**Figure 4:**
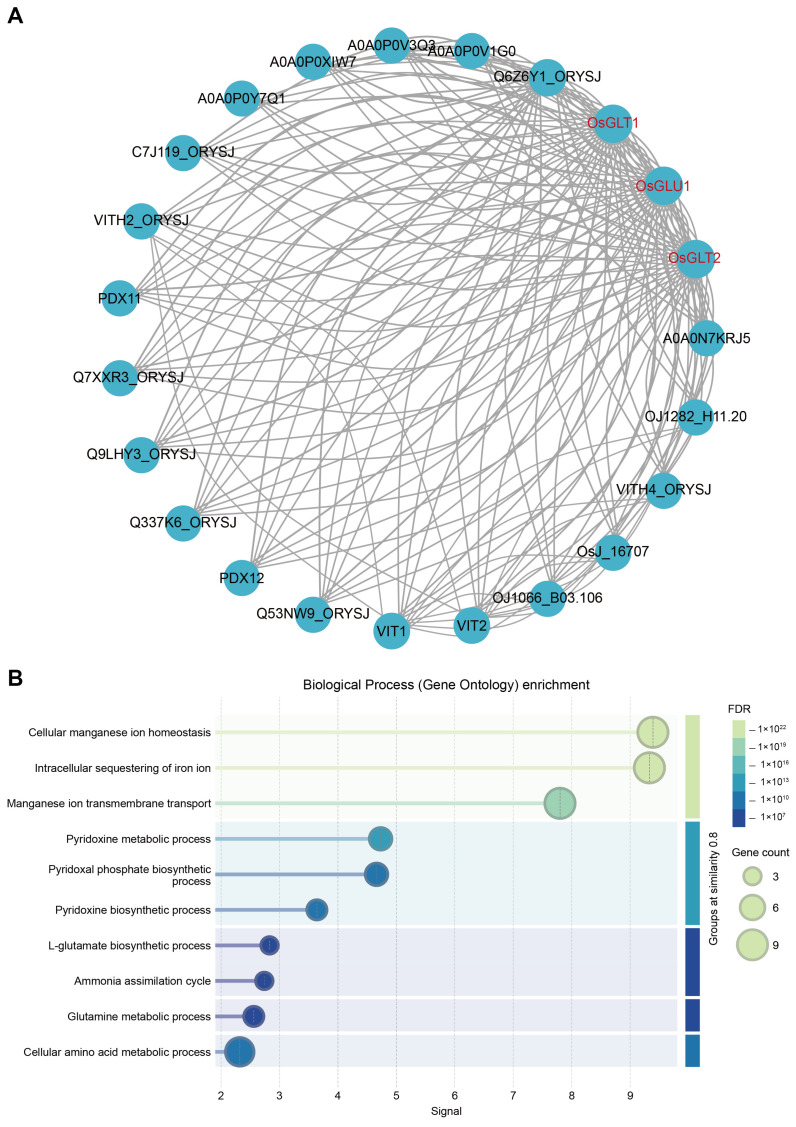
Interaction network and GO enrichment analysis of OsGOGAT proteins: (**A**) Protein–protein interaction (PPI) network of four OsGOGAT proteins (OsGLT1, OsGLT2, OsGLU1, highlighted in red). Gray lines represent the interaction relationships between GOGAT proteins and their partners. (**B**) GO biological process enrichment analysis of the interacting proteins was performed, with the x–axis indicating the signal value, the y–axis listing the enriched biological processes, the size of the circles representing the gene count, and the color gradient representing the false discovery rate (FDR).

Protein interactions play a crucial role in organisms. Proteins do this by forming complexes and building multi-protein networks, and they regulate a wide range of functions in organisms. Protein interactors of OsGOGAT family proteins were predicted using the STRING database. The results demonstrate that OsGOGAT proteins form a highly compact interaction network with ammonium transporters (AMT family), core nitrogen-assimilating glutamine synthetase (GS) family proteins, iron/manganese homeostasis regulators, and proteins involved in pyridoxal phosphate metabolism (PDX family). This indicates that beyond their canonical catalytic function as glutamine-α-oxoglutarate aminotransferases, GOGAT proteins are widely implicated in a broad spectrum of plant biological processes, including nitrogen uptake and translocation, micronutrient homeostasis maintenance, and cofactor biosynthesis (Figure 4A).

Gene Ontology (GO) enrichment analysis (Figure 4B) reveals that these interacting proteins are significantly enriched in biological processes spanning glutamine biosynthesis, inorganic NH_4_^+^ assimilation, divalent metal ion homeostasis, inorganic cation transmembrane transport, and pyridoxal phosphate biosynthesis. These findings collectively establish that OsGOGAT proteins, through synergistic interactions with diverse functional partners, serve as key regulatory nodes in the integration of critical physiological programs: central nitrogen metabolism, ionic homeostasis, and cofactor biosynthesis.

### 3.9. Secondary and Tertiary Structural Characterization of OsGOGAT Family Proteins

The secondary structures of three OsGOGAT family proteins were predicted using the online software SOPMA (http://npsa-pbil.ibcp.fr/cgi-bin/npsa_automat.pl?page=npsa_sopma.html) (accessed on 12 April 2026) (Figure S9A). All three OsGOGAT proteins are predominantly composed of α-helices and random coils, with extended strands as the secondary structural component. Specifically, the α-helix contents of OsGLT1, OsGLT2 and OsGLU1 are 36.59%, 37.71% and 41.24%, respectively; the random coil contents are 49.79%, 48.67% and 47.68%, respectively; and the extended strand contents are 13.61%, 13.62% and 11.08%. OsGLT1 and OsGLT2, both belonging to the GLT subfamily, exhibit highly similar proportions of each secondary structure type, reflecting intra-subfamily evolutionary conservation. In contrast, OsGLU1, the sole member of the GLU subfamily, has a markedly higher α-helix content and a relatively lower extended strand content than GLT subfamily members. This divergence in global secondary structure composition is tightly coupled to the differentiation in coenzyme binding properties, subcellular localization and catalytic function between the two subfamilies.

3D structures of OsGOGAT family proteins were predicted using AlphaFold2 (Figure S9B), with the resulting structural models visualized via PyMOL and individual conserved functional domains color-coded. The resolved 3D architectures exhibit robust concordance with the secondary structure prediction results. Further dissection of the secondary structure composition of each conserved functional domain reveals that the core catalytic modules of the GOGAT family—the GATase_2 domain, Glu_synthase domain, and GXGXG nucleotide-binding motif—share a highly conserved organizational pattern of α-helices and β-sheets across all three OsGOGAT proteins. Specifically, the GATase_2 domain uniformly consists of 16–18 α-helices and 15–16 β-sheets, the GXGXG motif comprises 6–7 α-helices and 10 β-sheets, and the Glu_synthase domain contains 18–20 α-helices and 7–8 β-sheets in all three proteins. These findings demonstrate that the spatial conformations of these core functional modules are highly stable, serving as the critical structural underpinning for the maintenance of GOGAT proteins’ canonical glutamine-α-oxoglutarate aminotransferase catalytic activity, and have been subject to strong purifying selection constraints throughout evolution.

Conversely, the Fer4_20 and Pyr_redox_2 domains, which mediate NADH binding and electron transfer, are exclusively present in OsGLT1 and OsGLT2 of the GLT subfamily, with nearly identical secondary structure compositions between the two paralogs (the Fer4_20 domain is uniformly composed of 8 α-helices, and the Pyr_redox_2 domain contains 12 α-helices and 13–14 β-sheets in both proteins). These two domains are completely absent in OsGLU1, the sole representative of the ferredoxin-dependent GLU subfamily, which also harbors significantly fewer α-helices and β-sheets within its Glu_syn_central domain compared with GLT subfamily members. This subfamily-specific domain architecture and secondary structure signature constitute the core molecular basis underlying the divergence in coenzyme dependency, subcellular localization, and biological function between the two GOGAT subfamilies.

### 3.10. Ka/Ks Analysis of OsGOGAT Genes

**Figure 5:**
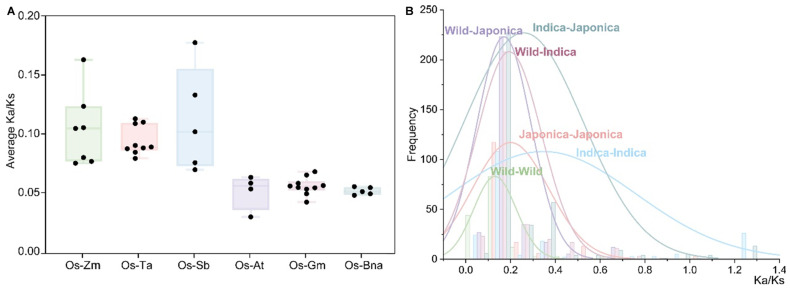
Ka/Ks Analysis of *GOGAT* Genes: Interspecific Comparisons Between *Oryza sativa* and Other Plant Species, and Intraspecific Comparisons Among Wild *Oryza*, *O. sativa* spp. *japonica* and *O. sativa* spp. *Indica*: (**A**) The evolutionary rates (Ka/Ks values) of *GOGAT* genes were calculated between *O. sativa* and six other species (*Zea mays*, *Triticum aestivum*, *Sorghum bicolor*, *Arabidopsis thaliana*, *Glycine max*, *Brassica napus*). The box plot visualizes the distribution of Ka/Ks ratios, with black dots representing individual gene pairs. The y-axis indicates the Ka/Ks value, and the x-axis shows the species comparison groups with *O. sativa* as the reference. (**B**) The *GOGAT* gene sequences from 8 wild *Oryza* accessions, 10 *O. sativa* spp. *japonica* cultivars, and 10 *O. sativa* spp. *indica* cultivars were collected to calculate the Ka/Ks ratios within and between groups. The main plot shows the distribution of Ka/Ks ratios for all the comparison groups, and the bar plot in the upper right corner displays the average Ka/Ks ratio of each group. The x-axis indicates the comparison groups, the y-axis represents the Ka/Ks ratio, and the dashed line at Ka/Ks = 1 marks the threshold of neutral evolution.

The non-synonymous substitution rate (Ka), synonymous substitution rate (Ks), and Ka/Ks ratios of *GOGAT* homologous gene pairs between *O. sativa* and other plant species were calculated using TBtools. All the interspecific pairwise comparisons yielded Ka/Ks values significantly less than 1 (Figure 5A), indicating that *GOGAT* genes have been subjected to strong purifying selection pressure throughout speciation and long-term evolutionary history, consistent with their functional conservation as core enzymes in plant nitrogen metabolism.

Further comparative analysis revealed that the mean Ka/Ks values of *GOGAT* homologous pairs between *O. sativa* and other Poaceae species (*T. aestivum*, *Z. mays*, *S. bicolor*) were significantly higher than those between *O. sativa* and three eudicot species (*A. thaliana*, *G. max*, *B. napus*). This finding indicates that *GOGAT* family genes have undergone relatively accelerated evolutionary rates within Poaceae, with weaker purifying selection constraints than those acting on the gene family during the divergence between monocot and eudicot lineages.

Integrated with the phylogenetic and synteny analyses performed in this study, the deep evolutionary divergence between monocot and eudicot lineages has allowed the *GOGAT* family to establish a stable framework of subfamily functional divergence and sequence conservation within each lineage. Critical amino acid residues within the core catalytic domains of GOGAT proteins are under extreme evolutionary constraint, which strictly restricts the accumulation of nonsynonymous substitutions, thus resulting in the lower Ka/Ks ratios observed in cross-lineage pairwise comparisons. In contrast, the relatively recent speciation events within the Poaceae family have permitted a modest accumulation of nonsynonymous substitutions in *GOGAT* genes during the diversification of Poaceae crops. These substitutions occur while the core catalytic function of the gene family is stringently maintained, and are likely driven by adaptive evolution to match the distinct developmental traits, nitrogen use efficiency (NUE) characteristics, and environmental adaptation demands of different Poaceae crops. Notably, *T. aestivum*, as an allohexaploid species, provides a more permissive evolutionary landscape for *GOGAT* genes via homoeolog subfunctionalization at the subgenome level following polyploidization, which further drives the elevated Ka/Ks ratios detected in within-Poaceae homologous gene pairs.

Collectively, purifying selection acts as the dominant evolutionary force shaping the *GOGAT* gene family across plant evolution, preserving the high functional conservation of its core biological roles. Divergence time between species, genome ploidy level, and species-specific environmental adaptation requirements collectively shape the divergent selection pressures acting on *GOGAT* genes across distinct species pairs. The relatively higher Ka/Ks ratios within the Poaceae family further provide a critical evolutionary framework for the subsequent identification of elite *GOGAT* allelic variants associated with high nitrogen use efficiency in cereal crops.

To dissect the selective and evolutionary signatures of the *GOGAT* gene family during *O. sativa* domestication, CDSs of *GOGAT* genes were collected from 8 wild *Oryza* accessions, 10 *O. sativa* spp. *japonica* varieties, and 10 *O. sativa* spp. *indica* varieties. The Ka/Ks ratio was systematically computed and profiled for homologous sequence pairs across 6 predefined groups: within wild *Oryza* (Wild-Wild), within japonica (Japonica-Japonica), within indica (Indica-Indica), between wild *Oryza* and japonica (Wild-Japonica), between wild *Oryza* and indica (Wild-Indica), and between indica and japonica (Indica-Japonica).

The mean Ka/Ks ratios of *GOGAT* gene pairs across all within-group and between-group comparisons were consistently less than 1 (Figure 5B), indicating that *GOGAT* genes are subject to strong purifying selection pressure in both wild and cultivated *O. sativa*, with their core nitrogen assimilation function highly conserved throughout the natural evolution and artificial domestication of *O. sativa*. Marked divergence in the distribution and mean values of Ka/Ks ratios was observed across the 6 groups, with mean values ranked in descending order as follows: indica-japonica between-group, indica within-group, wild *Oryza*-indica between-group, wild *Oryza*-japonica between-group, japonica within-group, and wild *Oryza* within-group.

Among these, the indica-japonica between-group comparison yielded the highest mean Ka/Ks ratio, which was significantly elevated relative to all the other groups, reflecting pronounced sequence divergence and markedly accelerated evolutionary rates of *GOGAT* genes following the split of the 2 cultivated *O. sativa* subspecies. The second-highest mean Ka/Ks ratio was observed in the indica within-group comparison, which was significantly higher than those in the japonica within-group and wild *Oryza* within-group, suggesting higher sequence polymorphism of *GOGAT* genes among indica varieties, coupled with weaker purifying selection constraints than those acting on japonica and wild *Oryza* populations. In contrast, the wild *Oryza* within-group comparison had the lowest mean Ka/Ks ratio, demonstrating that *GOGAT* genes in wild *Oryza* are the most sequence-conserved, subject to the strongest purifying selection pressure, and have maintained extreme stability of their core function during natural evolution.

For between-group comparisons of wild and cultivated *O. sativa*, the mean Ka/Ks ratio of the wild *Oryza*-indica group was significantly higher than that of the wild *Oryza*-japonica group, indicating a greater accumulation of nonsynonymous substitutions in *GOGAT* genes during indica domestication, with a more pronounced relaxation of purifying selection constraints than that observed during japonica domestication. This finding is fully consistent with the higher Ka/Ks ratios detected within the indica group. Furthermore, only a negligible number of sequence pairs with Ka/Ks ratios greater than 1 were detected across all the groups, with no statistically significant signals of positive selection identified. These results further confirm that purifying selection acts as the dominant evolutionary force shaping *GOGAT* genes during both natural evolution and artificial domestication of *O. sativa*, with no significant adaptive positive selection detected.

Collectively, *GOGAT* genes in *O. sativa* are globally subject to strong purifying selection constraints, with their core nitrogen assimilation function being highly conserved. The divergence between indica and japonica subspecies, as well as the artificial domestication of cultivated *O. sativa*, have shaped the differential landscape of selection pressures acting on *GOGAT* genes. Notably, the faster evolutionary rates and higher sequence polymorphism of *GOGAT* genes in the indica subspecies provide a potential reservoir of elite allelic variants for the genetic improvement of NUE in *O. sativa*.

### 3.11. Haplotype Diversity and Evolutionary Divergence of OsGOGAT Gene Family

To systematically characterize the genetic variation and domestication history of the *GOGAT* gene family, we performed comprehensive haplotype analysis of three functional members: *Ferredoxin-dependent GOGAT* (*Fd-GOGAT*) (*Os07g0658400*), *NADH-dependent GOGAT1* (*NADH-GOGAT1*) (*Os01g0681900*) and *NADH-dependent GOGAT2* (*NADH-GOGAT2*) (*Os05g0555600*). For each gene, we analyzed the 1.5 kb upstream promoter region and full-length CDS using high-quality resequencing data from 1990–2001 rice accessions, including ~1980 cultivated rice and 15–18 wild rice accessions retrieved from the MBKbase database. Notably, the three *GOGAT* family members exhibited strikingly distinct evolutionary patterns and domestication signatures, reflecting their divergent functional roles in rice nitrogen assimilation and source–sink regulation during reproductive growth (Table S3).

*Fd-GOGAT* displayed the most pronounced domestication signature among the three *GOGAT* genes. After strict quality filtering (removing accessions with >20% missing genotypes at the target locus), a total of 1990 high-quality accessions were retained for analysis, and 13 distinct haplotypes (Hap1–Hap13) were identified based on polymorphic sites in the promoter and CDS regions (Figure S10A). A clear domestication bottleneck was observed, with wild rice haplotypes forming a distinct cluster separate from cultivated rice haplotypes. Two dominant haplotypes accounted for 98.5% of all the cultivated rice accessions: Hap1 (*n* = 1187, 59.6%), which was almost exclusively restricted to temperate japonica and tropical japonica subspecies (96.2% japonica), and Hap2 (*n* = 784, 39.4%), which was predominantly enriched in indica and Basmati subspecies (92.7% indica/Basmati). This extreme complementary distribution pattern provides strong evidence that *Fd-GOGAT* underwent strong divergent selection during the independent domestication processes of indica and japonica rice. Phylogenetic analysis (Figure S10B) and haplotype network (Figure S10C) further confirmed this subspecific differentiation, with Hap1 and Hap2 separated by multiple mutational steps. Wild rice haplotypes were primarily clustered near the Hap2 branch, indicating that Hap2 represents the ancestral haplotype, while Hap1 was specifically selected and fixed in japonica populations during domestication. This strong subspecies differentiation suggests that *Fd-GOGAT* may play a key role in the nitrogen use efficiency differences between indica and japonica rice.

**Figure 6:**
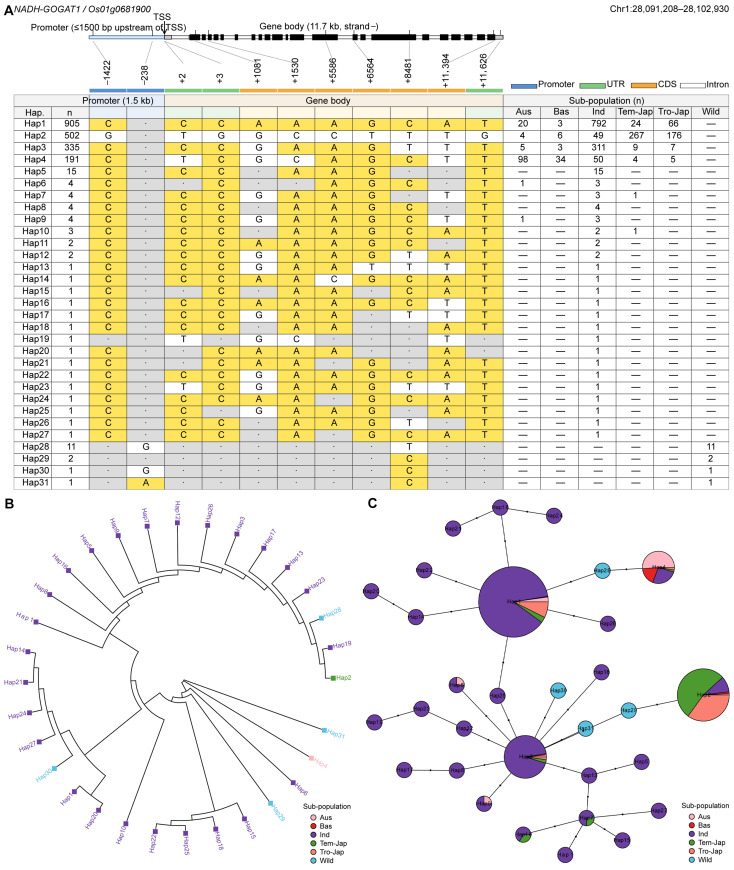
Haplotype analysis of *NADH-GOGAT1* in rice: (**A**) Haplotype polymorphism matrix of *NADH-GOGAT1* promoter and coding regions from 2001 rice accessions. Yellow-highlighted boxes indicate different nucleotides, and white boxes within letters indicate non-polymorphic nucleotide sites, and white boxes within black dots indicate no sequencing coverage. Ind, indica population. Tem-Jap, temperate japonica population. Tro-Jap, tropical japonica population. Bas, Basmati population. (**B**) Phylogenetic tree of the 31 haplotypes from 1986 cultivated and 15 wild rice accessions. Text on nodes = bootstrap %. Branches: cladogram (equal lengths). (**C**) *NADH-GOGAT1* haplotype network. The size of each circle is proportional to the sample count per haplotype. Black dots on the connecting lines denote mutational steps between different haplotypes.

*NADH-GOGAT1* was the most genetically diverse member of the *OsGOGAT* family. A total of 2001 high-quality accessions were analyzed, and 31 distinct haplotypes were identified, far exceeding the number observed for the other two *GOGAT* genes (Figure 6A). This high level of genetic diversity indicates that *NADH-GOGAT1* has accumulated abundant genetic variation during rice evolution. Hap1 was the dominant haplotype, accounting for 77.6% of all the cultivated rice accessions (*n* = 1553), and was widely distributed across all the rice subpopulations. However, it showed the highest frequency in temperate japonica (77.0%), followed by indica (68.5%) and tropical japonica (62.3%). The remaining 30 haplotypes were all low-frequency variants, with 12 haplotypes being unique to specific subpopulations and 5 haplotypes only present in wild rice. Phylogenetic analysis (Figure 6B) and haplotype network (Figure 6C) revealed that *NADH-GOGAT1* haplotypes did not form distinct subspecies-specific clades, but instead exhibited a complex reticulate evolutionary pattern. Multiple independent evolutionary branches were observed, with wild rice haplotypes interspersed among cultivated rice haplotypes. This complex evolutionary pattern suggests that *NADH-GOGAT1* may have undergone adaptive evolution in response to diverse environmental conditions, particularly varying nitrogen availability in different rice-growing regions.

A total of 1997 high-quality accessions were analyzed for *NADH-GOGAT2*, and 27 distinct haplotypes were identified (Figure S11A). Hap1 was the dominant haplotype, accounting for 77.8% of all the cultivated rice accessions (*n* = 1553), and was the predominant haplotype in indica (89.2%), temperate japonica (85.7%) and Basmati (91.9%) subpopulations. Notably, we identified a unique haplotype group consisting of Hap2–Hap7, which was almost exclusively restricted to tropical japonica accessions. These haplotypes accounted for 68.4% of all the tropical japonica accessions analyzed, but were present at frequencies of less than 2% in other subpopulations. Phylogenetic analysis (Figure S11B) and haplotype network (Figure S11C) showed that this tropical japonica-specific haplotype group formed a distinct evolutionary branch, separated from the main Hap1 branch by 4–6 mutational steps. Furthermore, these tropical japonica-specific haplotypes were more closely related to wild rice haplotypes than to Hap1, indicating that they represent an ancestral lineage that was specifically retained and selected in tropical japonica populations. This unique evolutionary pattern suggests that *NADH-GOGAT2* may have played an important role in the adaptation of tropical japonica rice to the specific soil nitrogen conditions and agricultural practices in tropical regions. The identification of these tropical japonica-specific haplotypes provides valuable genetic resources for improving nitrogen use efficiency in rice varieties adapted to tropical environments (Table S3).

### 3.12. Expression Profiles of OsGOGAT Family Genes in Source and Sink Tissues Under Different Nitrogen Levels

GOGAT serves as the core rate-limiting enzyme in the plant nitrogen assimilation pathway, and transcriptional regulation of its family genes directly determines plant nitrogen use efficiency and source–sink allocation balance. To systematically dissect the transcriptional response patterns of *OsGOGAT* family genes to varying nitrogen supply levels and elucidate the molecular mechanisms by which they mediate nitrogen remobilization, redistribution, and efficient material exchange between source and sink organs. High-throughput transcriptome sequencing analysis was performed to systematically characterize the expression profiles of three *OsGOGAT* family members (*OsGLT1*, *OsGLT2*, and *OsGLU1*) in major nitrogen source and sink tissues.

**Figure 7:**
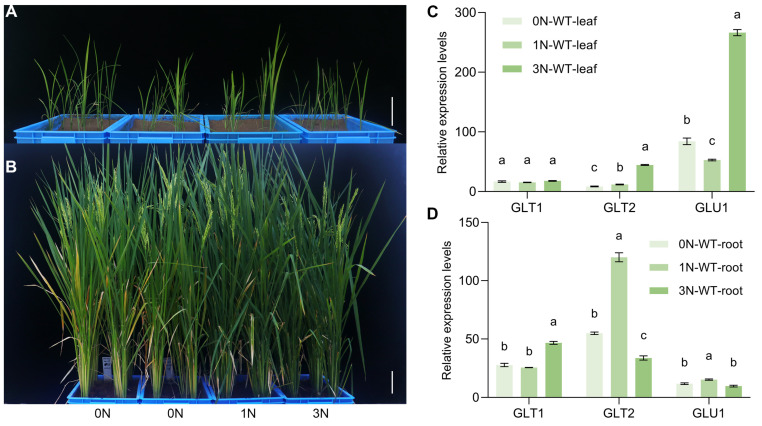
Transcriptional profiles of *GOGAT* genes in rice leaf and root tissues under three nitrogen gradient treatments: (**A**,**B**) All rice materials utilized in this study were in the Zhongxian 3037 genetic background. Plants were cultivated in a porous ceramic substrate with Kimura B nutrient solution. (**A**) Whole plants cultivated in standard Kimura B nutrient solution for 10 days at the seeding stage. Bar = 10 cm. (**B**) Whole rice plants were subjected to three nitrogen nutritional regimes (0N, 1N and 3N) for over 30 days following 50 days of vegetative growth in normal Kimura B nutrient solution. Two replicate pots of rice plants were set for the 0N treatment to meet the sampling requirements for plant material collection. Bar = 10 cm. (**C**) Expression dynamics of three *GOGAT* genes in leaf tissues during reproductive development (August–September), as determined by RNA-seq. Data are presented as mean ± SD (*n* = 3). Different letters denote statistically significant differences (*p* < 0.05). (**D**) Expression dynamics of three *GOGAT* genes in root tissues during reproductive development (August–September), as determined by RNA-seq analysis. Data are presented as mean ± SD (*n* = 3). Different letters denote statistically significant differences (*p* < 0.05).

The elite indica rice cultivar *O. sativa* cv. Zhongxian 3037, with uniform genetic background and stable agronomic traits, was used as the experimental material. Strict hydroponic experiments were conducted using modified Kimura B nutrient solution with three nitrogen treatments: 0N (N-deficient), 1N (moderate/optimal N), and 3N (high/excessive N) (Figure 7A,B). This experimental design possesses clear scientific and agronomic dual values: 0N simulates the nitrogen limitation conditions prevalent in low-input agricultural systems, enabling dissection of the molecular mechanisms underlying plant adaptation to low nitrogen stress; 1N represents the optimal nitrogen level for normal rice growth and development, serving as the baseline control for this study; 3N reflects the current status of excessive nitrogen application in intensive rice production in China, a practice that not only causes enormous waste of nitrogen resources but also leads to significantly reduced nitrogen use efficiency and a series of ecological and environmental problems. These three consecutive nitrogen gradients collectively constitute a systematic comparative research framework, which can comprehensively reveal the transcriptional response characteristics of rice nitrogen metabolism genes under different nitrogen supply levels and provide reliable experimental support for elucidating the molecular mechanisms by which exogenous nitrogen signals regulate source–sink nitrogen allocation.

In leaf tissues, *OsGLU1* exhibits the highest transcript abundance among the three *OsGOGAT* family genes under all nitrogen treatments (Figure 7C), consistent with its central role in photorespiratory NH_4_^+^ reassimilation and photosynthetic nitrogen fixation. 0N significantly suppresses *OsGLT2* expression, while 1N and 3N markedly upregulate its transcription, showing a clear nitrogen dose-dependent transcriptional induction characteristic. In contrast, *OsGLT1* transcript levels are unaffected by external nitrogen supply levels, reflecting its stable housekeeping function in leaf nitrogen metabolism.

The expression pattern in root tissues shows significant tissue-specific differentiation from that in leaves (Figure 7D). *OsGLT1* and *OsGLT2* are the predominantly expressed isoforms in roots. *OsGLT1* displays a high nitrogen-induced expression characteristic in roots, with expression levels peaking under 3N treatment and significantly higher than those under 0N and 1N treatments. *OsGLT2* expression is significantly inhibited by both nitrogen deficiency and excess nitrogen. This result further confirms that *OsGLT2* is the key functional gene mediating primary nitrogen assimilation in rice roots. *OsGLU1* is constitutively expressed at low levels in roots and is essentially unregulated by nitrogen supply. This opposite nitrogen response pattern between roots and leaves reveals tissue-specific functional differentiation of the *OsGOGAT* family: under low nitrogen conditions, roots preferentially activate *GLT* subfamily genes to mediate primary NH_4_^+^ assimilation, while *OsGLU1* constitutively performs photosynthetic nitrogen assimilation functions in leaves (Table S4).

**Figure 8:**
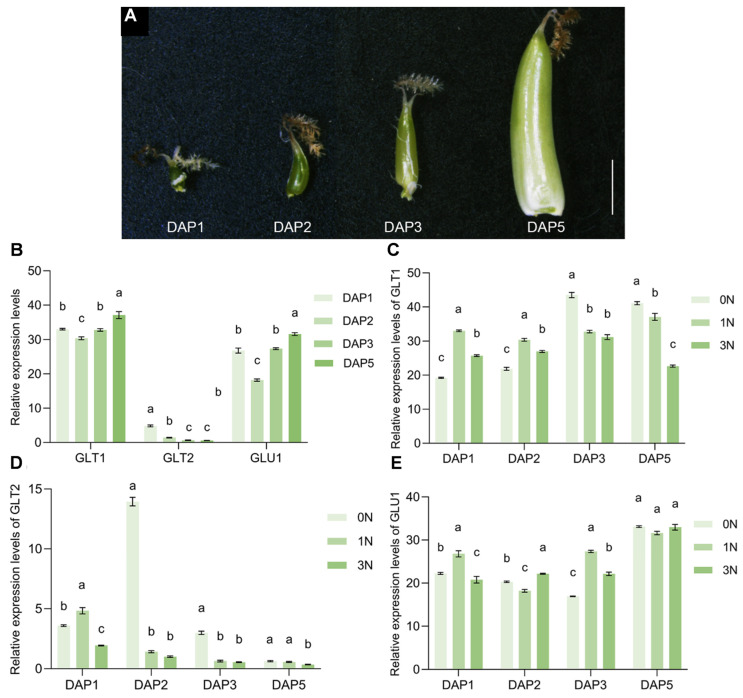
Expression pattern analysis of GOGAT genes in rice young immature embryos subjected to three nitrogen gradient treatments: (**A**) Morphological characteristics of rice young embryos at four developmental stages (1, 2, 3–4, and 5–6 DAP). Bar = 3 mm. (**B**) Expression dynamics of three GOGAT genes in rice young embryos during reproductive development (August–September) under normal nitrogen (1N) condition, as determined by RNA-seq. Bars correspond to mean ± SD (*n* = 3). Different letters denote statistically significant differences (*p* < 0.05). (**C**–**E**) Expression dynamics of three GOGAT genes in rice young embryos treated with three nitrogen nutritional conditions (0N, 1N, and 3N), as determined by RNA-seq. Bars correspond to mean ± SD (*n* = 3). Different letters denote statistically significant differences (*p* < 0.05).

To clarify the expression characteristics and nitrogen regulatory patterns of *OsGOGAT* genes during the initiation of rice grain filling. Rice young embryos were collected at four consecutive developmental stages: 1, 2, 3–4, and 5–6 days after pollination (DAP) (Figure 8A), and the expression dynamics of *OsGOGAT* genes during young embryo development under different nitrogen supply levels were analyzed. The developmental expression profile under 1N conditions shows that the transcript levels of *OsGLT1* and *OsGLU1* genes first decrease and then gradually increase along with young embryo development, reaching expression peaks at 5–6 DAP. In contrast, *OsGLT2* exhibits extremely low overall expression abundance throughout the four developmental stages of young embryos and gradually decreases with embryo development (Figure 8B).

Expression analysis under different nitrogen levels reveals distinct nitrogen response characteristics among the three *OsGOGAT* genes (Figure 8C–E). *OsGLT1* shows significantly higher expression levels under 0N and 1N treatments than under 3N treatment at all developmental stages. Under 0N and 3N conditions, expression levels continuously increase and then decrease, peaking at 3 DAP, while under 1N conditions, expression levels first decrease and then gradually increase, peaking at 5 DAP. High expression is observed under low nitrogen levels during the latter two stages of embryodevelopment (Figure 8C). *OsGLT2* shows extremely low overall expression, with detectable expression only at 2 DAP, and exhibits strong nitrogen deficiency induction characteristics, with almost undetectable expression in other developmental stages. At 2 and 3–4 DAP, transcript abundance under 0N conditions is significantly higher than that under 1N and 3N treatments, indicating that its expression is negatively correlated with external nitrogen availability during early embryogenesis. Expression levels remain low at 3–4 and 5–6 DAP, with no significant difference between 1N and 3N treatments at 3–4 DAP and no significant difference between 0N and 1N treatments at 5–6 DAP (Figure 8D). *OsGLU1* is highly expressed at all developmental stages and generally upregulated along with development. The most significant difference is observed at 3 DAP, while no statistically significant difference exists among the three nitrogen treatments at 5 DAP (Table S5).

Collectively, all three target genes initiate expression at the early stage of rice young embryo development, yet their spatiotemporal expression patterns diverge significantly with marked differences in expression levels and dynamic profiles. *OsGLT1* and *OsGLU1* are the dominant members in young embryos, maintaining high expression throughout early embryogenesis and showing a clear continuous upward trend as embryo differentiation and grain matter accumulation progress. In contrast, *OsGLT2* remains at an overall low expression level with a distinct dynamic: it exhibits a transient expression peak at the onset of young embryo development, followed by continuous downregulation along with development. Physiologically, 3–5 DAP is the active period of starch synthesis and accumulation in rice grains. During grain filling, grain nitrogen supply relies heavily on remobilization and reuse of nitrogen stored in vegetative tissues, which contributes 80% of total grain nitrogen accumulation and serves as the primary nitrogen source at this stage [53,54,55]. *OsGLT1* expression peaks first at 3 DAF, while *OsGLU1* peaks at 5 DAF. Their expression peaks align well with the dynamic demands of sustained nitrogen assimilation, amino acid metabolism and storage substance synthesis during grain filling, indicating that *OsGLT1* and *OsGLU1* play critical biological roles in nitrogen reuse and assimilation as well as endosperm matter accumulation during rice grain filling. These findings provide fundamental data for further dissecting the molecular mechanisms underlying nitrogen-mediated regulation of grain filling and yield formation in rice.

### 3.13. Tissue-Specific Nitrogen Responses of GOGAT Enzyme Activity and Glutamic Acid (Glu) Accumulation in Rice Source and Sink Tissues

**Figure 9:**
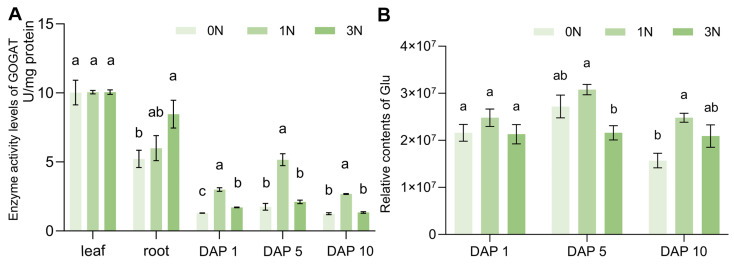
GOGAT enzyme activity and Glu contents analysis in source and sink tissues of wild-type rice under different nitrogen concentrations. 0N, nitrogen-free treatment; 1N, normal nitrogen treatment (control); 3N, high nitrogen treatment. Source tissue: leaf; sink tissues: root and embryo at 1, 5 and 10 days after pollination (DAP): (**A**) Enzyme activity levels of GOGAT. (**B**) Contents of Glu in young embryos at DAP1, DAP5 and DAP10. Enzyme activity assays and determination of metabolites were performed with three independent biological replicates, each containing three technical replicates. Values are expressed as mean ± standard error (SE). Different letters denote statistically significant differences (*p* < 0.05).

To comprehensively investigate the regulatory effects of nitrogen availability on nitrogen assimilation in source and sink tissues during rice reproductive growth, we quantified the activity of GOGAT, the rate-limiting enzyme downstream of GS in the GS/GOGAT cycle, and the accumulation of its catalytic product Glu in flag leaves (photosynthetic source), roots (nitrogen uptake source), and developing embryos (reproductive sink) at 1, 5, and 10 DAP under three nitrogen regimes (0N; 1N; 3N) (Figure 9).

Our data revealed that GOGAT activity displayed a distinct tissue-specific regulatory pattern in response to external nitrogen supply (Table S6). In flag leaves, GOGAT activity remained constitutively high and showed no significant difference among the three nitrogen treatments (all *p* > 0.05, Figure 9A), indicating that leaf nitrogen assimilation is buffered against fluctuations in external nitrogen availability to maintain stable photosynthetic function. In roots, the primary organ for inorganic nitrogen uptake, GOGAT activity was positively correlated with nitrogen concentration: activity under 1N and 3N conditions was 1.2-fold and 1.6-fold higher than that under 0N, respectively (*p* < 0.05). In sharp contrast, in developing embryos, both 0N and 3N significantly inhibited GOGAT activity compared to the optimal 1N condition at all three developmental stages examined. This result suggests that embryo nitrogen metabolism is tightly regulated within a narrow physiological nitrogen concentration range to ensure normal seed development. Notably, GOGAT activity exhibited a consistent developmental trajectory in young embryos regardless of nitrogen supply, with activity levels peaking at 5 DAP and declining significantly by 10 DAP. This synchronized developmental pattern with GS provides strong biochemical evidence that 5 DAP represents the metabolically most active stage of nitrogen assimilation during rice embryo development, coinciding with the completion of aleurone layer differentiation and the initiation of rapid storage compound accumulation in the endosperm.

Consistent with the changes in GOGAT enzyme activity, the accumulation of Glu, the direct product of GOGAT catalysis, also showed significant developmental dynamics and nitrogen-dependent regulation in young embryos (Figure 9B). Under the 1N, Glu content followed the same trend as GOGAT activity, reaching the highest level at 5 DAP, which was 1.3-fold and 1.5-fold higher than that at 1 DAP and 10 DAP, respectively. This strong positive correlation between enzyme activity and product accumulation confirms the central role of GOGAT in controlling glutamate biosynthesis during rice seed development. Nitrogen availability differentially affected Glu accumulation at different developmental stages. At 1 DAP, no significant difference in Glu content was observed among the three nitrogen treatments (all *p* > 0.05), indicating that early embryo development is less sensitive to external nitrogen fluctuations due to maternal nutrient supply. At 5 DAP, however, 3N significantly reduced Glu content by 28.7% compared to 1N (*p* < 0.05), while 0N showed a non-significant decreasing trend. At 10 DAP, 0N led to a 42.1% reduction in Glu content compared to 1N (*p* < 0.05), whereas 3N had no significant effect. These stage-specific responses suggest that the regulatory mechanisms of glutamate metabolism shift during embryo development, with the mid-developmental stage (5 DAP) being most sensitive to excess nitrogen and the late stage (10 DAP) being most vulnerable to nitrogen deficiency (Table S7).

Taken together, these results demonstrate that GOGAT-mediated glutamate biosynthesis is tightly regulated in a tissue-specific and developmental stage-dependent manner in response to nitrogen availability. The coordinated regulation of GOGAT activity and Glu accumulation ensures the maintenance of nitrogen homeostasis in different tissues and supports normal rice seed development under varying nitrogen conditions.

## 4. Discussion

### 4.1. Evolutionary Conservation and Functional Divergence of the GOGAT Gene Family Across Monocot and Dicot Plants

Glutamate synthase (GOGAT) is a core enzyme in the GS/GOGAT cycle, which is responsible for the primary assimilation of inorganic nitrogen in plants [20]. Our comprehensive genome-wide analysis of *GOGAT* genes across 12 monocot and dicot species revealed that the *GOGAT* gene family can be divided into two ancient and evolutionarily conserved subfamilies: *GLU* (*Fd-GOGAT*) and *GLT* (*NADH-GOGAT*). This classification is consistent with previous studies in Arabidopsis and rice, indicating that the functional divergence of Fd-dependent and NADH-dependent GOGAT isoforms occurred early in the evolution of land plants. Notably, we observed a unique subfunctionalization event within the *GLT* subfamily in Poaceae species, which further diverged into *GLT1* and *GLT2* subgroups [56,57,58]. This monocot-specific duplication event suggests that *GOGAT* genes have undergone adaptive evolution to meet the higher nitrogen demand of cereal crops during their domestication and improvement. Collinearity analysis showed that *GOGAT* genes have maintained high synteny between rice and other cereal crops, particularly maize and wheat, indicating that these genes have retained essential functions throughout grass evolution. The non-synonymous substitution rate (Ka)/synonymous substitution rate (Ks) analysis further confirmed that all the *GOGAT* genes have been subjected to strong purifying selection during evolution, with Ka/Ks values consistently less than 1. This strong selective constraint reflects the essential role of GOGAT in plant nitrogen metabolism. However, we also detected slightly higher Ka/Ks values in the *NADH-GOGAT* subfamily compared to the *Fd-GOGAT* subfamily, suggesting that *NADH-GOGAT* genes have experienced more relaxed selective pressure and may have evolved more diverse functions in different plant species.

### 4.2. Distinct Domestication Signatures of OsGOGAT Genes Revealed by Haplotype Analysis of 3000 Rice Accessions

The natural genetic variation in *GOGAT* genes provides valuable resources for improving nitrogen use efficiency (NUE) in rice. Our haplotype analysis of three *OsGOGAT* genes using nearly 3000 rice accessions revealed strikingly distinct evolutionary patterns and domestication signatures, reflecting their divergent functional roles in rice adaptation to different nitrogen environments. *Fd-GOGAT* exhibited the strongest domestication signature among the three *GOGAT* genes, with two dominant haplotypes showing extreme complementary distribution between indica and japonica subspecies. Wild rice haplotypes were primarily clustered near the indica-dominant Hap2 branch, indicating that Hap2 represents the ancestral haplotype, while the japonica-dominant Hap1 was specifically selected and fixed during japonica domestication. This strong divergent selection suggests that *Fd-GOGAT* may be a key genetic locus underlying the differences in NUE between indica and japonica rice. In contrast, *NADH-dependent GOGAT1* (*NADH-GOGAT1*) showed the highest haplotype diversity, with 31 distinct haplotypes and no clear subspecies-specific differentiation. This complex evolutionary pattern suggests that *NADH-GOGAT1* has undergone adaptive evolution in response to diverse environmental conditions, particularly varying nitrogen availability in different rice-growing regions. Most interestingly, we identified a unique haplotype group of *NADH-dependent GOGAT2* (*NADH-GOGAT2*) that is almost exclusively restricted to tropical japonica accessions. These tropical japonica-specific haplotypes are more closely related to wild rice haplotypes, indicating that they represent an ancestral lineage that was specifically retained and selected in tropical japonica populations to adapt to the specific soil nitrogen conditions in tropical regions.

### 4.3. Tissue-Specific and Developmental Stage-Dependent Regulation of GOGAT-Mediated Nitrogen Homeostasis During Reproductive Growth

Reproductive growth is the most critical period for determining rice grain yield and quality, and efficient nitrogen transport and assimilation in source and sink tissues during this stage directly affect grain nitrogen accumulation. Our multi-tissue and multi-stage analysis revealed a sophisticated regulatory network of GOGAT-mediated nitrogen assimilation that ensures nitrogen homeostasis under varying nitrogen conditions. The distinct nitrogen response patterns of GOGAT activity in different tissues reflect their specialized functional roles in the whole-plant nitrogen economy. The constitutive high activity of GOGAT in flag leaves regardless of external nitrogen supply demonstrates that photosynthetic nitrogen assimilation is a top priority for plants, even under nitrogen deficiency [59]. This buffer mechanism ensures stable carbon fixation and provides sufficient carbon skeletons for nitrogen assimilation and transport to developing grains. In contrast, the positive correlation between root GOGAT activity and nitrogen supply reflects the dynamic regulation of primary nitrogen uptake in response to soil nitrogen availability. The most striking finding is the strict regulation of GOGAT activity in developing embryos within a narrow nitrogen concentration range. Both nitrogen deficiency and excess significantly inhibited GOGAT activity, indicating that embryo development is extremely sensitive to nitrogen status. This strict regulation is essential for ensuring normal embryo development and preventing abnormal seed formation caused by nitrogen imbalance. Furthermore, the consistent peak of GOGAT activity and glutamate accumulation at 5 days after pollination (DAP) across all the nitrogen treatments identifies this stage as the metabolic switch point during rice seed development, when nitrogen assimilation shifts from supporting embryo differentiation to fueling rapid storage compound accumulation in the endosperm.

### 4.4. Characterization of OsGOGAT Genes Uncovers Elite Genetic Resources for Molecular Breeding of High-NUE and High-Yield Rice

Characterization of evolutionary divergence, haplotype variation, and developmental regulation of *OsGOGAT* genes highlights their promising potential in molecular breeding for enhanced NUE and grain productivity. Markedly differentiated *Fd-GOGAT* haplotypes between indica and japonica subspecies, with japonica-dominant Hap1 under strong domestication selection, serve as robust markers for marker-assisted selection to optimize photorespiratory nitrogen reassimilation across diverse cultivation environments [53]. *NADH-GOGAT1*, featuring high haplotype diversity and a non-redundant function in root primary ammonium assimilation, represents a prime target for mining elite natural alleles to improve low-nitrogen uptake efficiency, sustaining active tillering and panicle formation while reducing nitrogen fertilizer input [54]. Tropical japonica-specific *NADH-GOGAT2* haplotypes, critical for leaf nitrogen remobilization during grain filling, provide unique genetic resources for breeding rice adapted to tropical soil nitrogen regimes, boosting spikelet number per panicle and grain filling efficiency. Tight developmental control of GOGAT activity at the 5-day post-pollination metabolic switch enables fine-tuning of embryo nitrogen homeostasis for stable seed development and balanced grain protein accumulation [60,61]. These gene resources lay a solid foundation for molecular design breeding to synergistically improve rice NUE and yield.

Nevertheless, several notable limitations exist in the current study. Direct in vivo experimental validation is still lacking for the causal roles of *GOGAT* isoforms and elite haplotypes in NUE and grain development, their upstream regulators, interacting partners and subcellular localization. Further functional genomic studies are required to corroborate these inferences and support translational rice NUE breeding.

## 5. Conclusions

This study performed genome-wide characterization of the *GOGAT* gene family across 12 monocot and dicot species, combined with haplotype analysis of nearly 3000 rice accessions and multi-level functional validation under three nitrogen regimes, systematically revealing the evolutionary history, natural genetic variation and regulatory mechanisms of GOGAT-mediated nitrogen assimilation in rice. Cross-species comparative analysis showed that the *GOGAT* family originated from two ancient paralogous copies predating the monocot-dicot split. A unique Poaceae-specific duplication event in the *GLT* subfamily provided the genetic basis for the high nitrogen demand of cereal crops, with all the members under strong purifying selection. Haplotype analysis uncovered distinct domestication trajectories of three *OsGOGAT* genes. The subspecies-specific haplotypes of *Fd-GOGAT* and the tropical japonica-specific haplotype group of *NADH-GOGAT2* offer valuable molecular markers for nitrogen-efficient rice breeding. Functional analysis demonstrated tissue-specific and developmental stage-dependent regulation of GOGAT activity during reproductive growth, and identified 5 DAP as the critical metabolic switch point for seed nitrogen metabolism, providing a scientific basis for precise nitrogen fertilizer application. These findings lay a foundation for understanding rice nitrogen assimilation, and future work will focus on functional validation of the elite *GOGAT* haplotypes.

## Data Availability

The original contributions presented in this study are included in the article/Supplementary Materials. The raw data for RNA-seq of source and sink tissue under different nitrogen treatments can be accessed from the NCBI SRA database (accession number: PRJNA1473341). Further inquiries can be directed to the corresponding authors.

## Supplementary Materials

The following supporting information can be downloaded at https://www.mdpi.com/article/10.3390/genes17070791/s1, Figure S1: Chromosomal localization of *rice glutamate synthase* (*OsGOGAT*) family genes; Figure S2: Gene structure analysis of *OsGOGAT* genes; Figure S3: Prediction of transmembrane domains and signal peptides of OsGOGAT proteins; Figure S4: Three-dimensional structural models of OsGOGAT proteins; Figure S5: Amino acid sequence alignment of ferredoxin–dependent glutamate synthase (GLU) proteins across diverse plant species; Figure S6: Amino acid sequence alignment of NADH–dependent glutamate synthase (GLT) proteins across diverse plant species; Figure S7: Conserved motif analysis of GOGAT proteins from multiple plant species; Figure S8: HMM logos of conserved motifs in GOGAT proteins; Figure S9: Prediction of secondary and tertiary structures of three OsGOGAT family proteins in *O. sativa*; Figure S10: Haplotype analysis of *Fd-GOGAT* in rice; Figure S11: Haplotype analysis of *NADH-GOGAT2* in rice; Table S1: Information about *GOGAT* family genes in multiple plant species; Table S2: *GOGAT* genes in 28 Oryza species; Table S3: Whole-genome SNP Genotyping Dataset of *GOGAT* gene Family in 3000 Rice Accessions; Table S4: RNA-seq data of *GOGAT* genes in roots and flag leaves; Table S5: RNA-seq data of *GOGAT* genes in embryos. Table S6: Data of GOGAT enzyme activity in embryos; Table S7: Contents of GLU in embryos.

## Abbreviations

The following abbreviations are used in this manuscript:

ABA	Abscisic acid
AMT	Ammonium transporter
ANOVA	Analysis of variance
ATP	Adenosine triphosphate
BLAST	Basic Local Alignment Search Tool
CDD	Conserved Domain Database
CDSs	Coding sequences
DAP	Days after pollination
FDR	False discovery rate
Fd-GOGAT	Ferredoxin-dependent GOGAT
Gln	Glutamine
GLT	NADH-dependent GOGAT
GLU	Ferredoxin-dependent GOGAT
Glu	Glutamic acid
GO	Gene Ontology
GOGAT	Glutamate synthase
GS	Glutamine synthetase
HMM	Hidden Markov model
HSD	Honestly significant difference
Itol	Interactive Tree of Life
Ka	Non-synonymous substitution rate
Ks	Synonymous substitution rate
MeJA	Methyl jasmonate
ML	Maximum likelihood
MSA	Multiple sequence alignment
N	Nitrogen
NADH-GOGAT	NADH-dependent GOGAT
NADH-GOGAT1	NADH-dependent GOGAT1
NADH-GOGAT2	NADH-dependent GOGAT2
NCBI	National Center for Biotechnology Information
NUE	Nitrogen use efficiency
PDX	Pyridoxal metabolism-related protein
Pfam	Protein Family Database
RNA-seq	RNA sequencing
PPI	Protein–protein interaction
SMART	Simple Modular Architecture Research Tool
SOPMA	Self-Optimized Prediction Method with Alignment
STRING	Search Tool for the Retrieval of Interacting Genes/Proteins
TAIR	The Arabidopsis Information Resource
TBtools	Toolbox for Biologists
UPGMA	Unweighted Pair Group Method with Arithmetic Mean
UTR	Untranslated region
WT	Wild Type
Zoops	Zero-or-one-occurrence-per-sequence
3D	Three-dimensional
