# Supplementary material for: Genomic Evolution and Nitrogen Response Analysis of Glutamate Synthase Gene Family in Rice Source–Sink Tissues During Grain Filling
Source: Genes (Basel). 2026 Jul 12;17(7):791. doi: 10.3390/genes17070791 (PMC13408792; doi:10.3390/genes17070791)
Supplement: Supplementary file 1 [file genes-17-00791-s001.zip › genes-4398465 Supplementary Figures.pdf]

Article

Genomic Evolution and Nitrogen Response Analysis of *Glutamate Synthase* Gene Family in Rice Source-Sink Tissues During Grain Filling

Shuai Fu[1], Zixin Xiang [1], Yuelin Wu [1], Huihui Zhang [1], Haiting Hu [1], Zhuocheng Liu [2,*] and Han Yang [1,*]

[1] Anhui Province Key Laboratory of Plant Resources and Biology, Anhui Engineering Research Center for Green Production Technology of Drought Grain Crops, College of Life Sciences, Huaibei Normal University, Huaibei 235000, China; 17356152707@163.com (S.F.); 19733887713@163.com (Z.X.); 17756987437@163.com (Y.W.); 19733889023@163.com (H.Z.); hht112119@163.com (H.H.)

[2] School of Biological and Food Engineering, Suzhou University, Suzhou 234000, China

* Correspondence: liuzhuocheng@ahszu.edu.cn (Z.L.); yanghan@chnu.edu.cn (H.Y.)

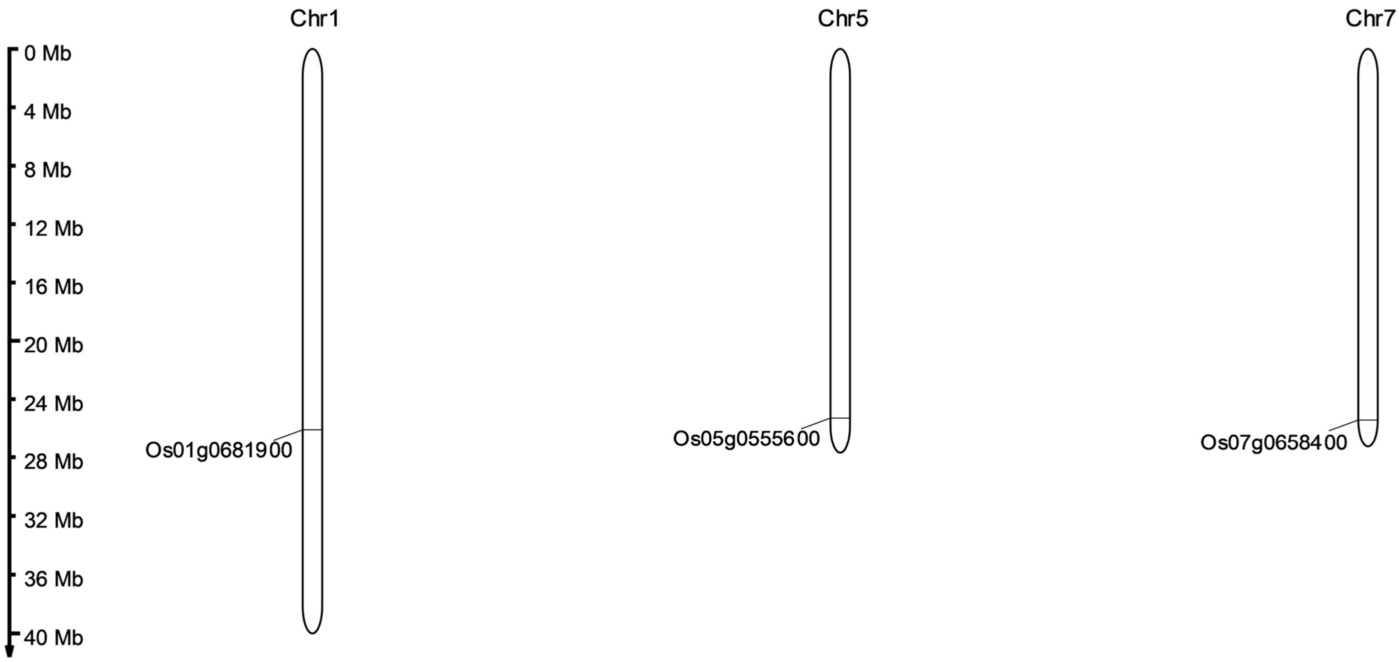

Figure S1. Chromosomal localization of *rice glutamate synthase* (*OsGOGAT*) family genes. Vertical bars represent rice chromosomes, with the scale on the left indicating physical position in megabases (Mb). The three *OsGOGAT* genes are mapped to their respective chromosomes, with horizontal lines marking their precise locations and gene IDs labeled beside.

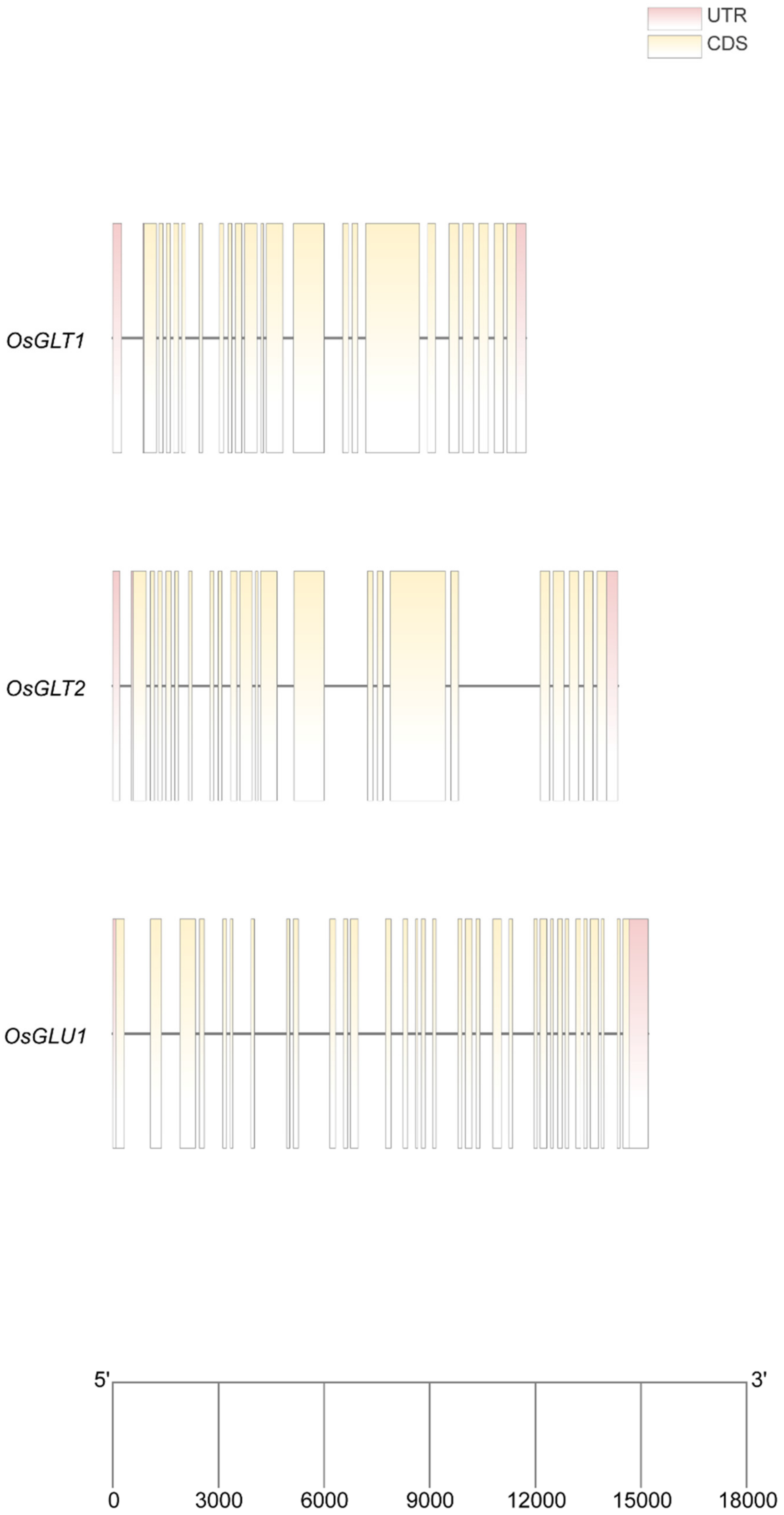

Figure S2. Gene structure analysis of *OsGOGAT* genes. The exon-intron structure of four *OsGOGAT* genes (*OsGLT1*, *OsGLT2*, and *OsGLU1*) was visualized. Pink regions represent untranslated regions (UTRs), and yellow regions represent coding sequences (CDSs). The scale bar at the bottom indicates the nucleotide length (bp) from the 5' to 3' end of the genes.

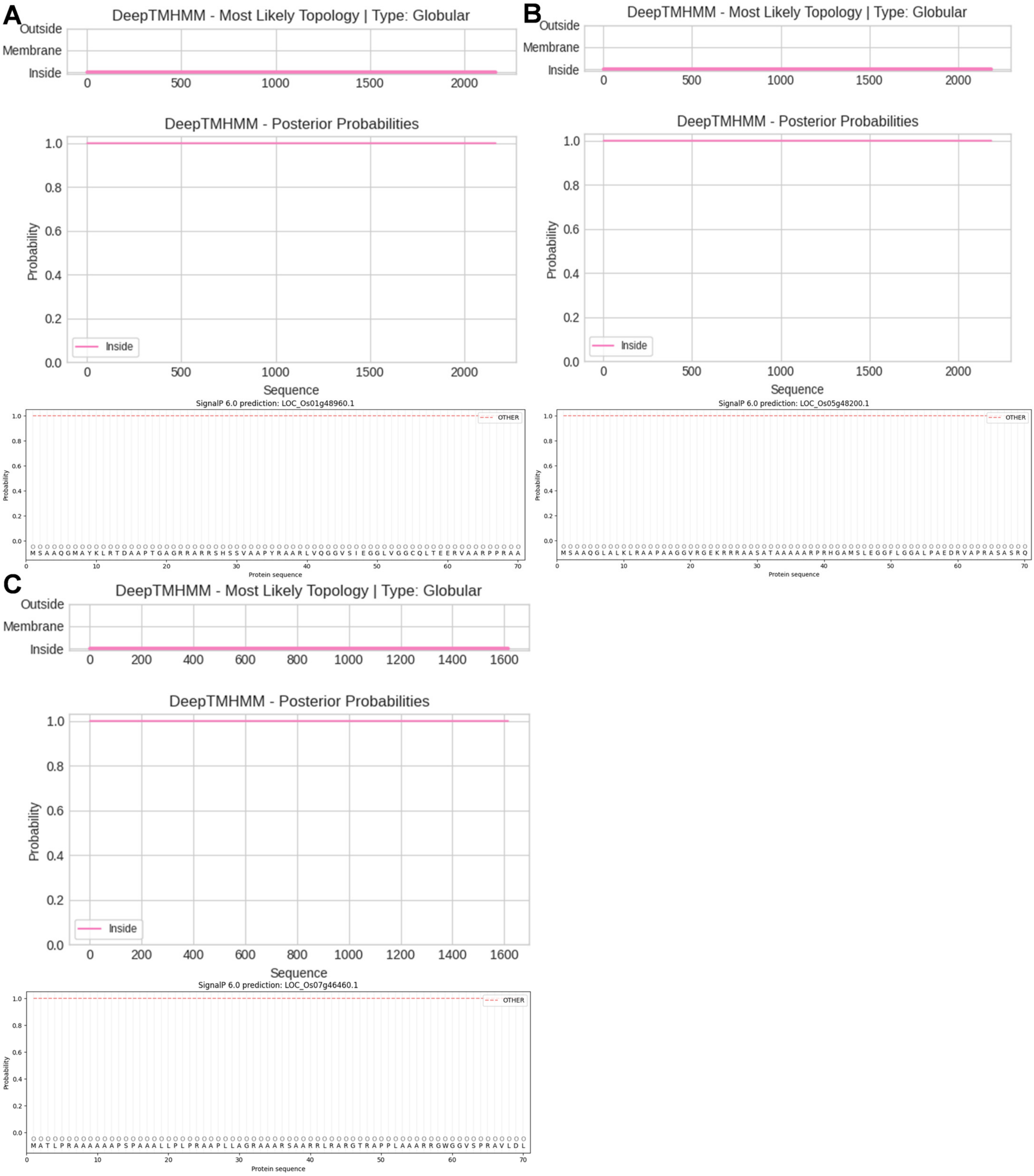

Figure S3. Prediction of transmembrane domains and signal peptides of OsGOGAT proteins. Transmembrane helices and signal peptides of three OsGOGAT proteins (OsGLT-1, OsGLT-2, and OsGLU-1). (**A**–**C**) The upper panels in each column show the most likely topology and posterior probabilities predicted. All three proteins were predicted to be globular proteins with no transmembrane helices, and their full-length sequences were localized inside the cell with 100% confidence. The lower panels display the signal peptide prediction results, indicating that none of the three proteins contained a signal peptide, with all residues classified as "Other" at a probability of 1.0. These results suggest that OsGOGAT proteins are soluble intracellular proteins that are neither membrane-localized nor targeted to the canonical secretory pathway.

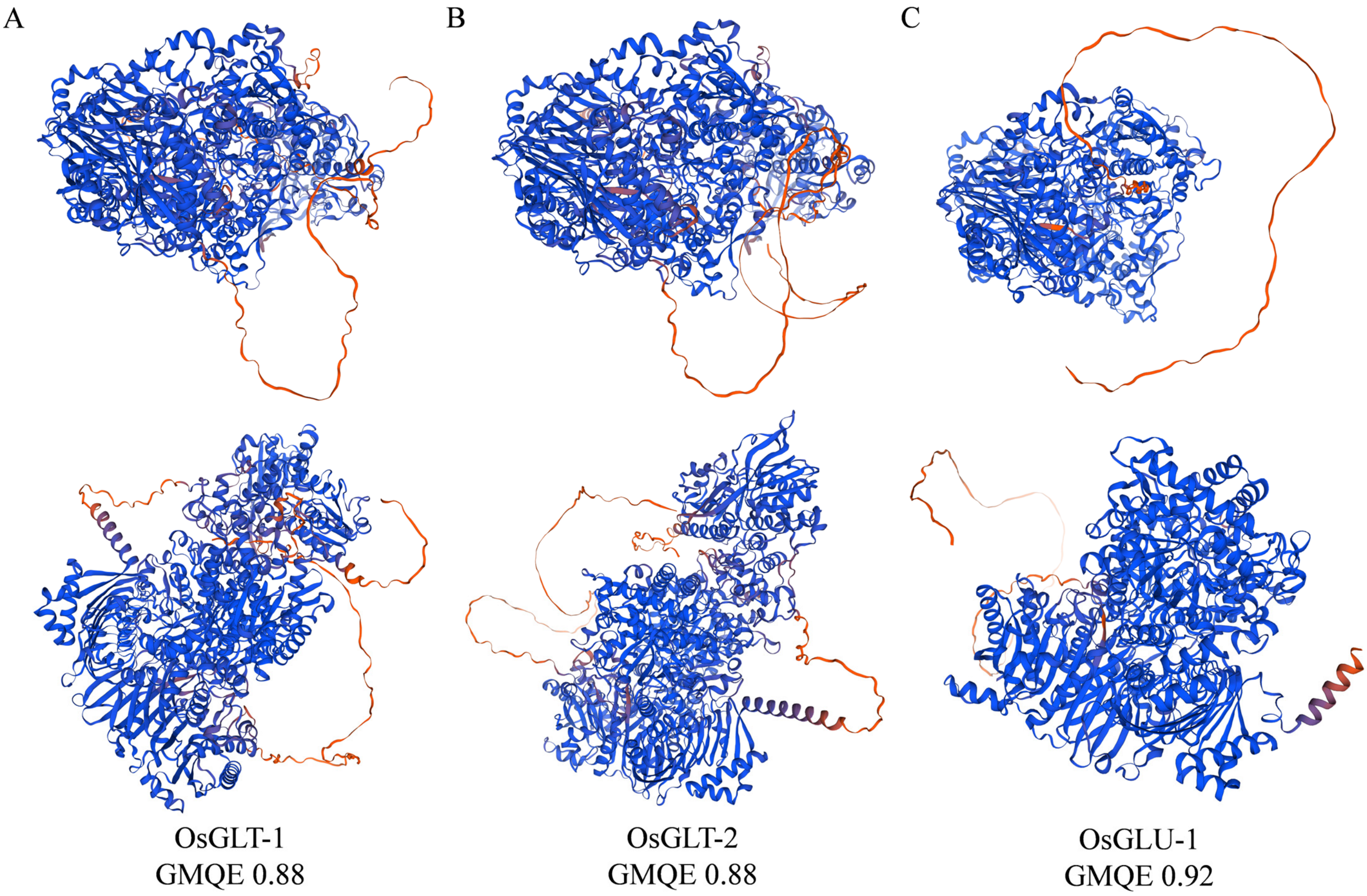

Figure S4. Three-dimensional structural models of OsGOGAT proteins. (**A**-**C**) The three-dimensional structures of three OsGOGAT proteins (OsGLT-1, OsGLT-2, and OsGLU-1) were modeled using SWISS-MODEL. Each protein is visualized in two orthogonal views: the front view of the monomeric structure is shown in the top row, and the top-down view is presented in the bottom row. The overall quality of all models was evaluated using the Global Model Quality Estimate (GMQE) metric. All models exhibited high structural reliability, with their secondary structure elements predominantly consisting of α-helices and random coils, confirming the high evolutionary conservation of the monomeric assembly pattern of OsGOGAT proteins.

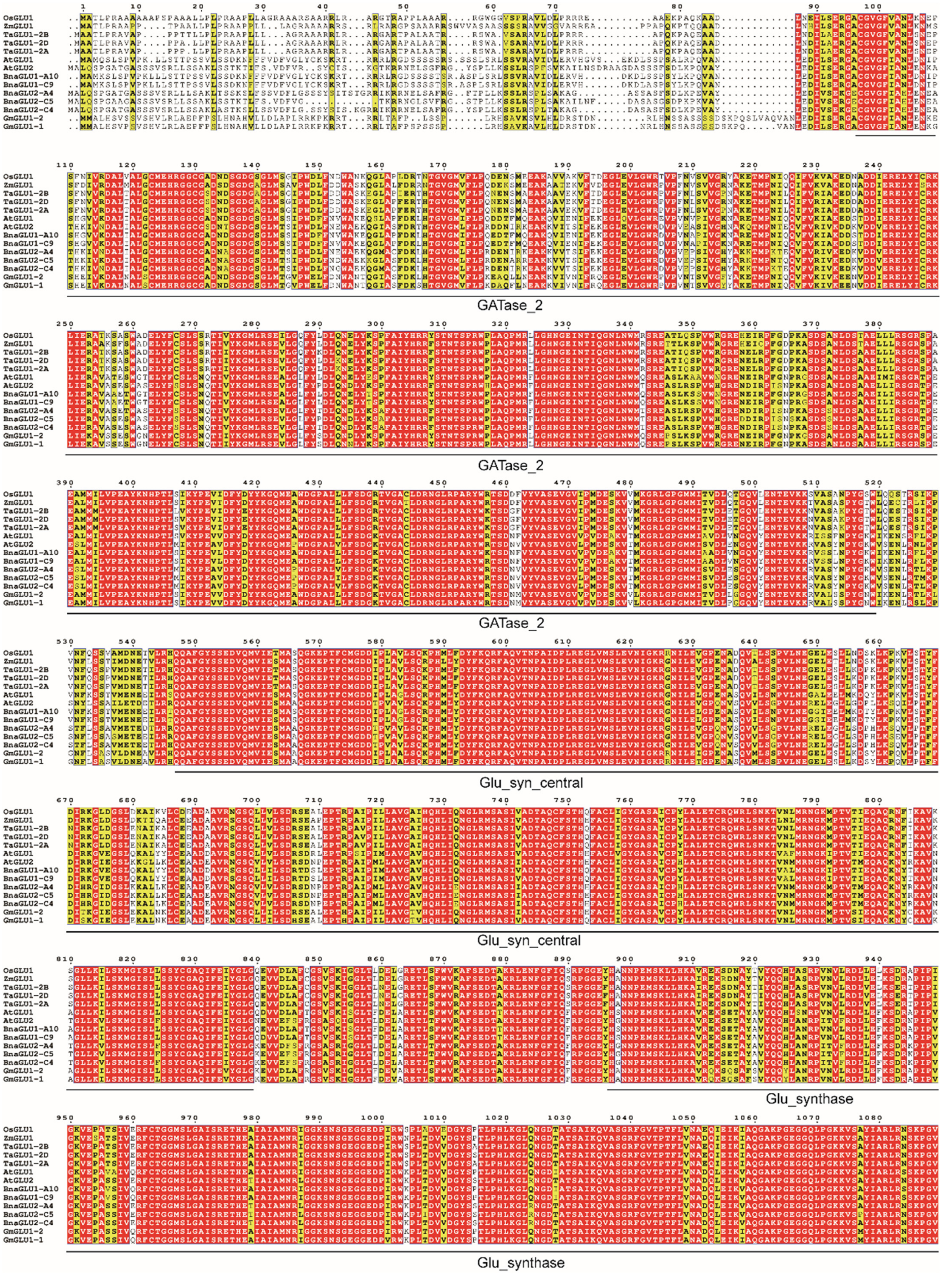
OsGLU1
ZmGLU1
TaGLU1-2B
TaGLU1-2D
TaGLU1-2A
AtGLU1
AtGLU2
BnaGLU1-A10
BnaGLU1-C9
BnaGLU2-A4
BnaGLU2-C5
BnaGLU2-C4
GmGLU1-2
GmGLU1-1
GATase_2
GATase_2
GATase_2
Glu_syn_central
Glu_syn_central
Glu_synthase
Glu_synthase

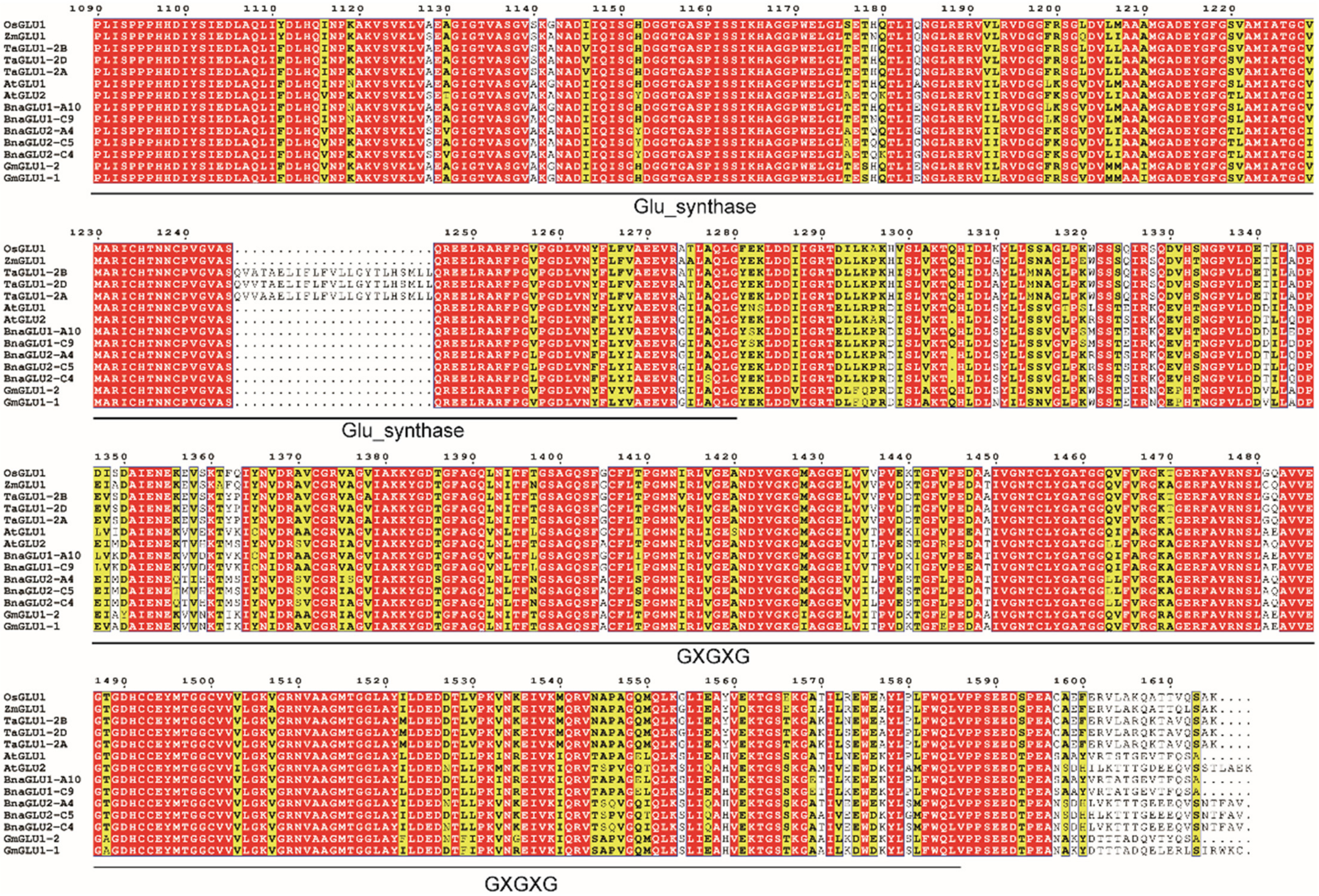

Figure S5. Amino acid sequence alignment of ferredoxin-dependent glutamate synthase (GLU) proteins across diverse plant species. Multiple sequence alignment of GLU proteins from *Oryza sativa*, *Triticum aestivum*, *Zea mays*, *Arabidopsis thaliana*, *Glycine max*, and *Brassica napus*. Conserved amino acid residues are highlighted in red (100% identity) and yellow (high similarity), with variable regions shown in gray. The conserved functional domains of GOGAT proteins, including GATase_2, Glu_syn_central, Glu_synthase, and GXGXG, are labeled above the corresponding sequence regions.

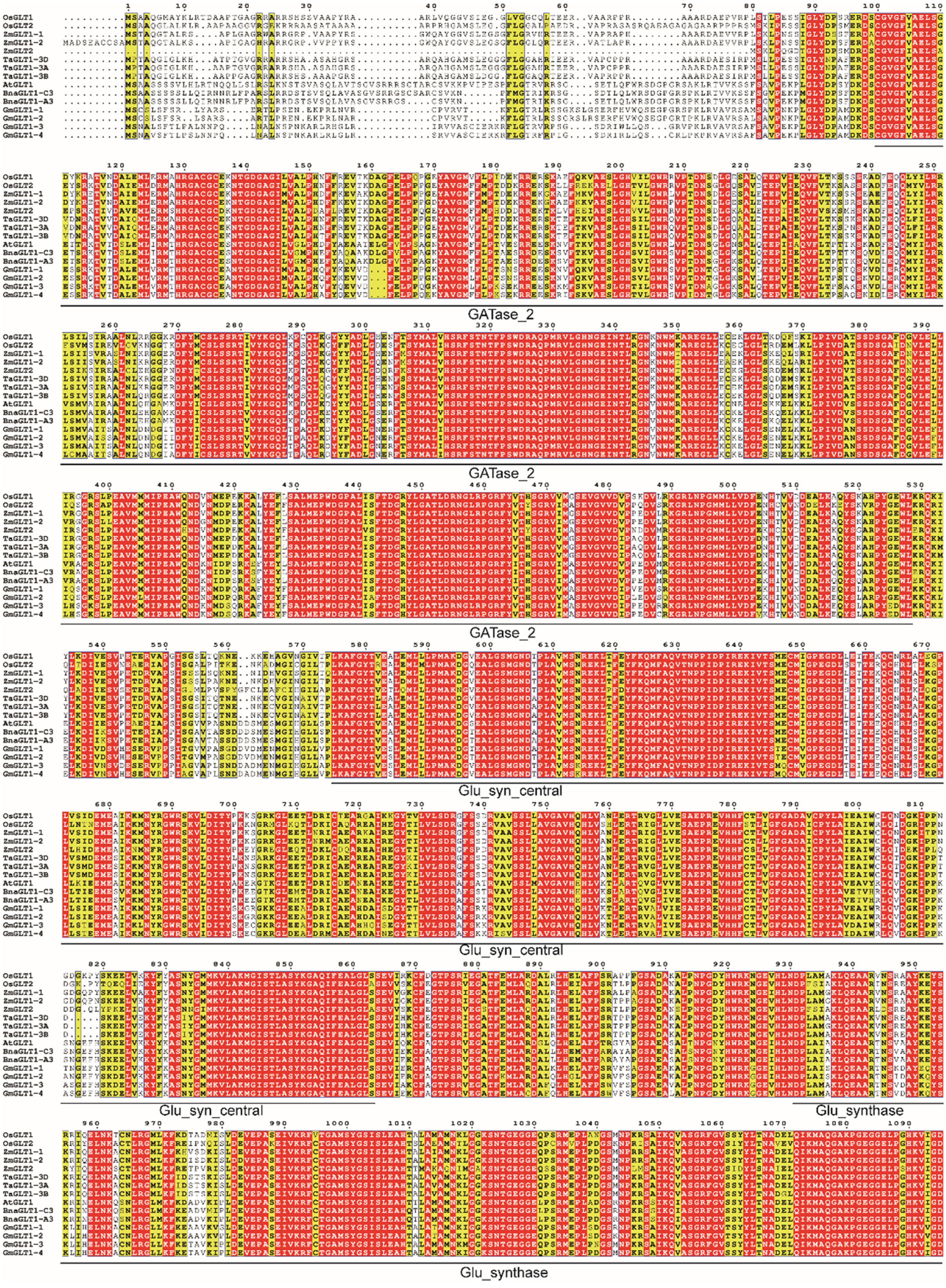
OsGLT1
OsGLT2
ZmGLT1-1
ZmGLT1-2
ZmGLT2
TaGLT1-3D
TaGLT1-3A
TaGLT1-3B
AtGLT1
BnaGLT1-C3
BnaGLT1-A3
GmGLT1-1
GmGLT1-2
GmGLT1-3
GmGLT1-4
GATase_2
GATase_2
GATase_2
Glu_syn_central
Glu_syn_central
Glu_syn_central
Glu_synthase
Glu_synthase

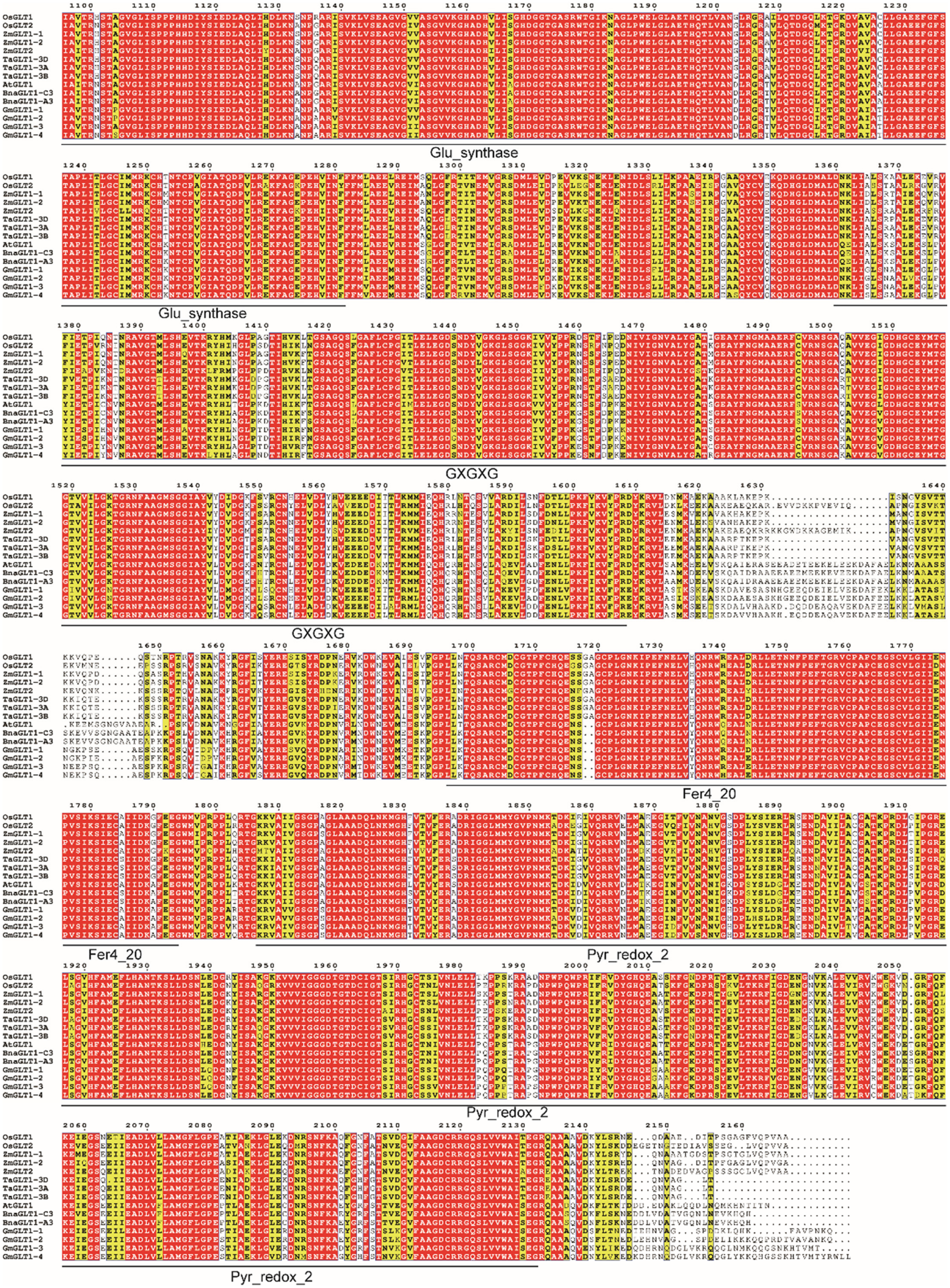

Figure S6. Amino acid sequence alignment of NADH-dependent glutamate synthase (GLT) proteins across

diverse plant species. Multiple sequence alignment of GOGAT proteins from *O. sativa*, *T. aestivum*, *Z. mays*, *A. thaliana*, *G. max*, and *B. napus*. Conserved amino acid residues are highlighted in red (100% identity) and yellow (high similarity), with variable regions shown in gray. The ferredoxin-binding related domains Fer4_20 and Pyr_redox_2, as well as the core catalytic domains of GOGAT, are labeled above the corresponding sequence regions.

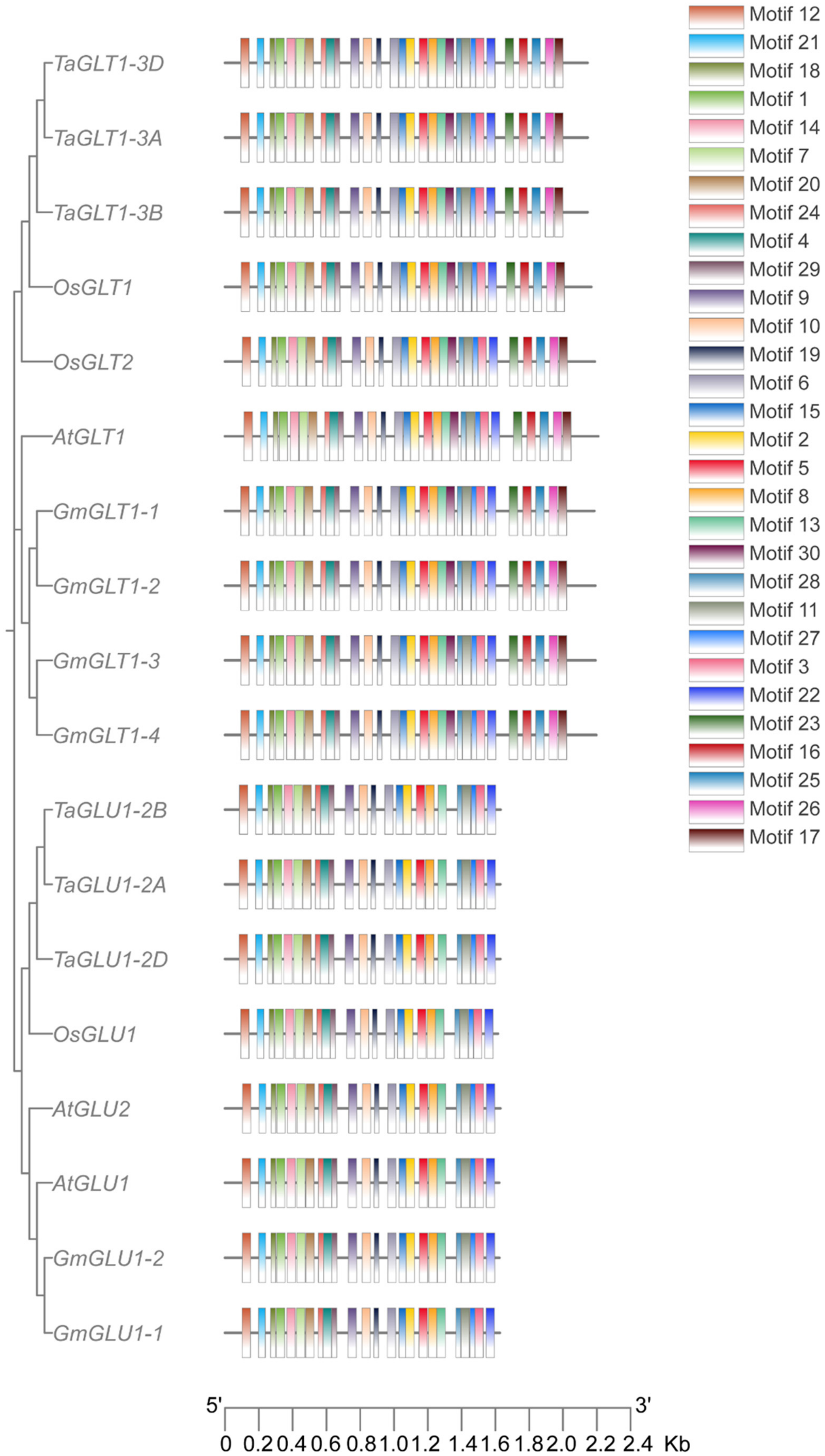

Figure S7. Conserved motif analysis of GOGAT proteins from multiple plant species. Conserved motifs in GOGAT proteins from *O. sativa*, *T. aestivum*, *A. thaliana*, and *G. max*. A total of 30 conserved motifs (Motif 1–30) were detected, with distinct colored boxes representing different motifs. The scale bar at the bottom indicates the amino acid length of the proteins

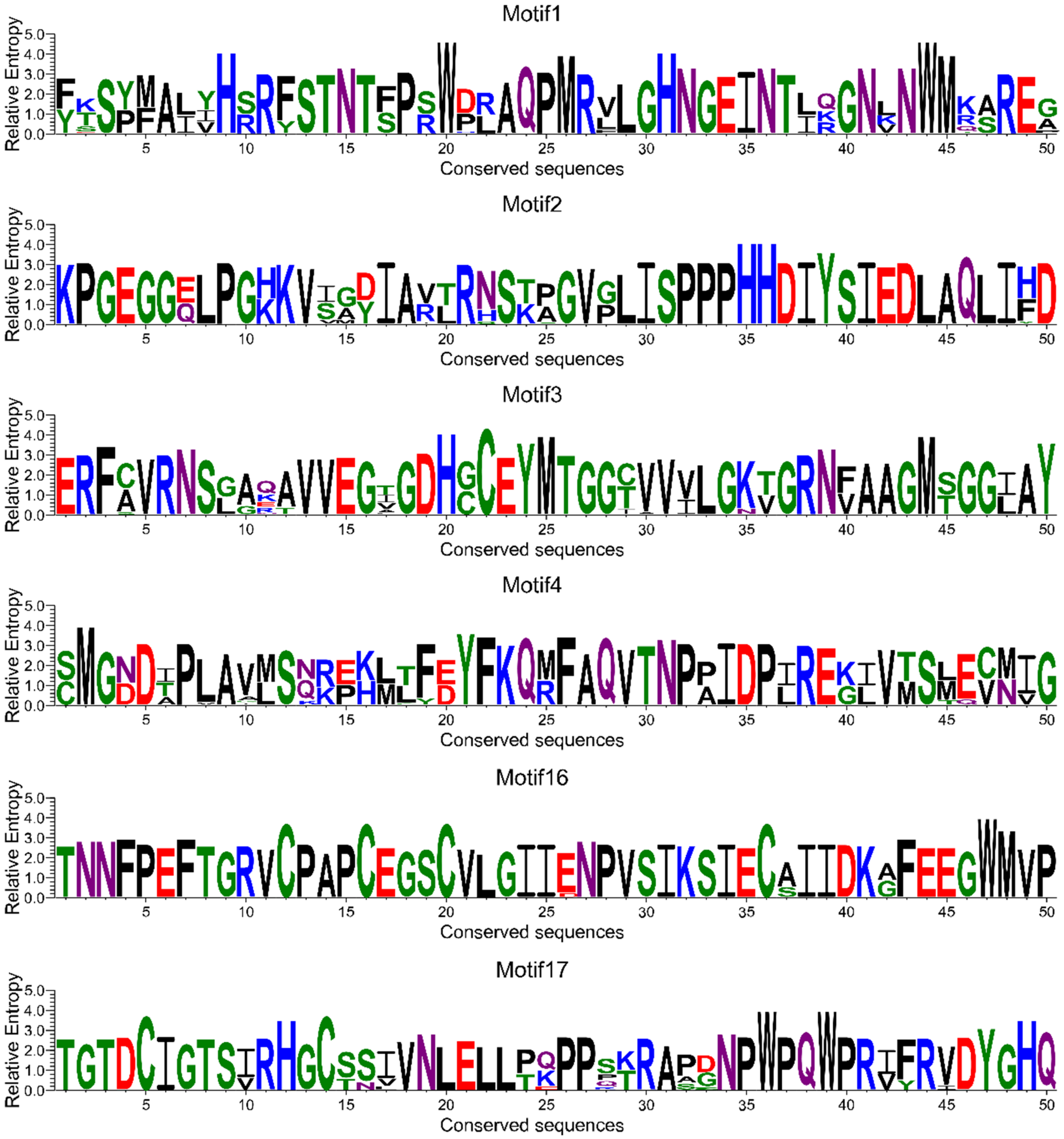

Figure S8. HMM logos of conserved motifs in GOGAT proteins. Sequence logos of three conserved motifs (Motif 1, Motif 2, Motif 3, Motif 4, Motif 16 and Motif 17) from GOGAT proteins were generated to visualize the amino acid conservation at each position. The X-axis represents the conserved amino acid sequences of each motif, while the Y-axis indicates the relative entropy, which reflects the conservation rate of each residue.

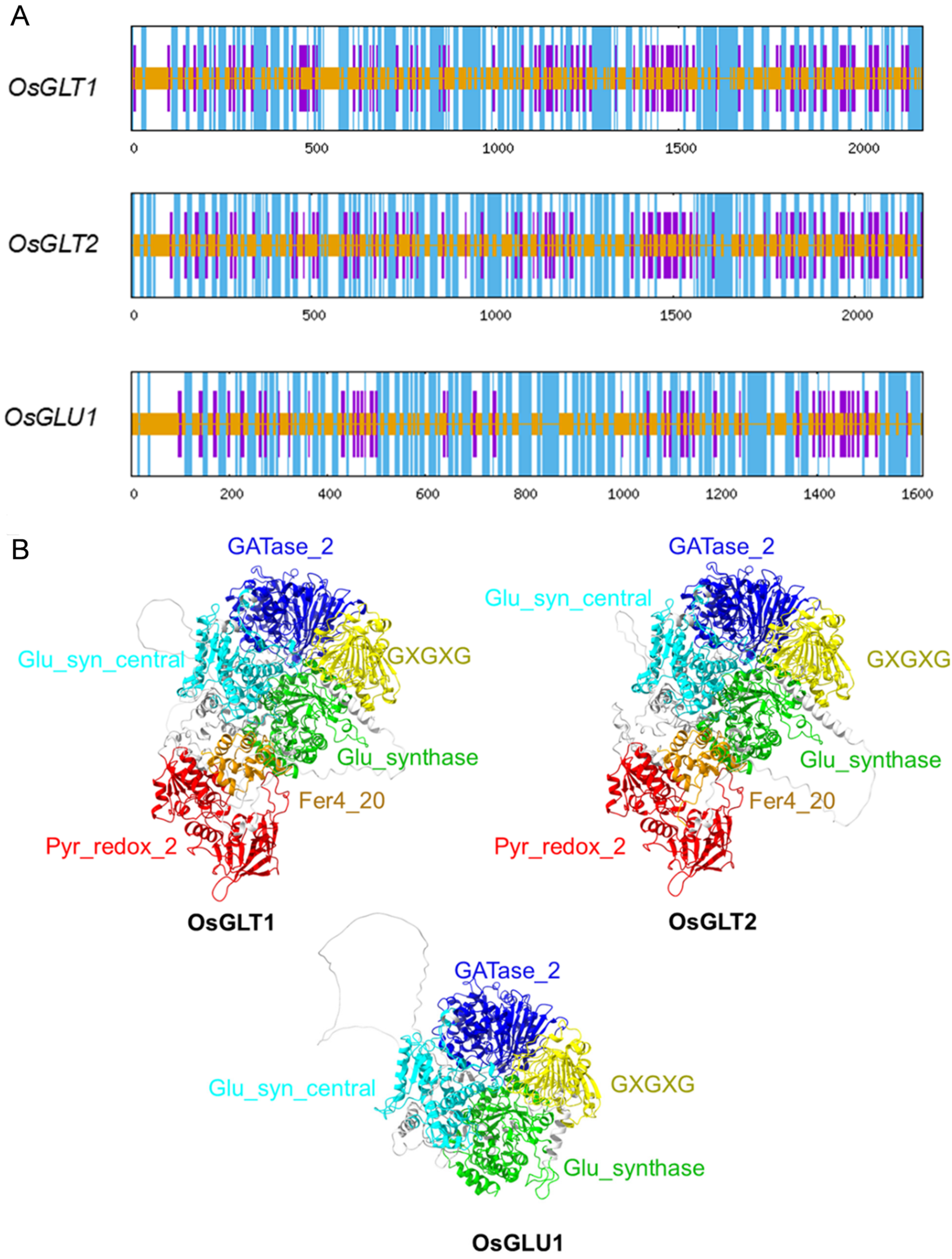

Figure S9. Prediction of secondary and tertiary structures of three OsGOGAT family proteins in *O. sativa*. (**A**) Secondary structure prediction of three OsGOGAT proteins. Blue bars represent α-helices, purple bars represent extended strands, and yellow bars represent random coils. (**B**) Predicted tertiary structures of three OsGOGAT family proteins. Different colors in the structures correspond to distinct conserved functional domains of GOGAT proteins, including the GATase_2 domain, Glu_synthase domain, Glu_syn_central domain, and ferredoxin-binding related domains (Fer4_20 and Pyr_redox_2).

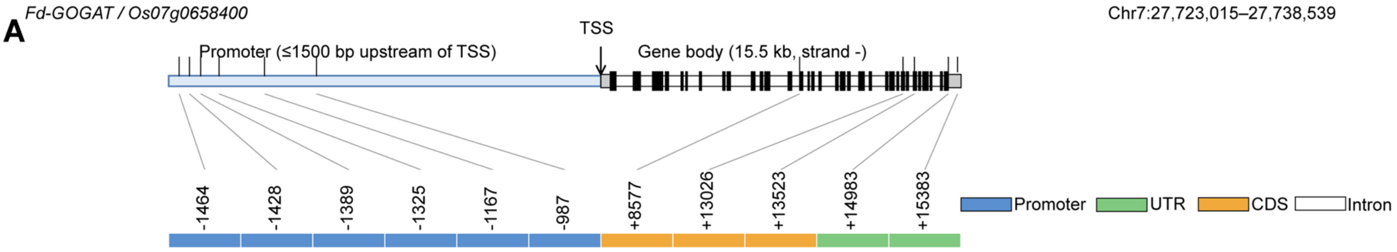

		Promoter (1.5 kb)						Gene body					Sub-population (n)					
Hap.	n												Aus	Bas	Ind	Tem-Jap	Tro-Jap	Wild
Hap1	1187	A	G	C	A	G	T	T	T	G	G	A	15	32	1056	36	48	—
Hap2	784	A	A	A	T	A	C	T	T	T	A	T	113	13	176	270	205	7
Hap3	2	A	G	C	A	·	T	T	T	G	G	A	—	—	2	—	—	—
Hap4	1	·	·	C	A	·	T	T	T	·	G	A	—	—	1	—	—	—
Hap5	1	·	·	C	A	·	·	T	·	·	·	A	—	—	1	—	—	—
Hap6	1	A	G	C	A	G	T	T	T	T	G	A	—	—	1	—	—	—
Hap7	1	A	G	C	A	A	T	T	T	G	G	A	—	—	1	—	—	—
Hap8	1	·	·	·	A	G	·	·	T	G	·	·	—	—	1	—	—	—
Hap9	1	A	G	·	·	G	T	T	T	·	G	A	—	—	1	—	—	—
Hap10	5	T	T	A	A	G	A	G	G	T	C	G	—	—	—	—	—	5
Hap11	3	A	T	A	A	G	A	G	G	T	C	G	—	—	—	—	—	3
Hap12	2	·	T	A	A	G	A	G	G	T	C	G	—	—	—	—	—	2
Hap13	1	·	T	A	A	G	A	·	G	T	C	G	—	—	—	—	—	1

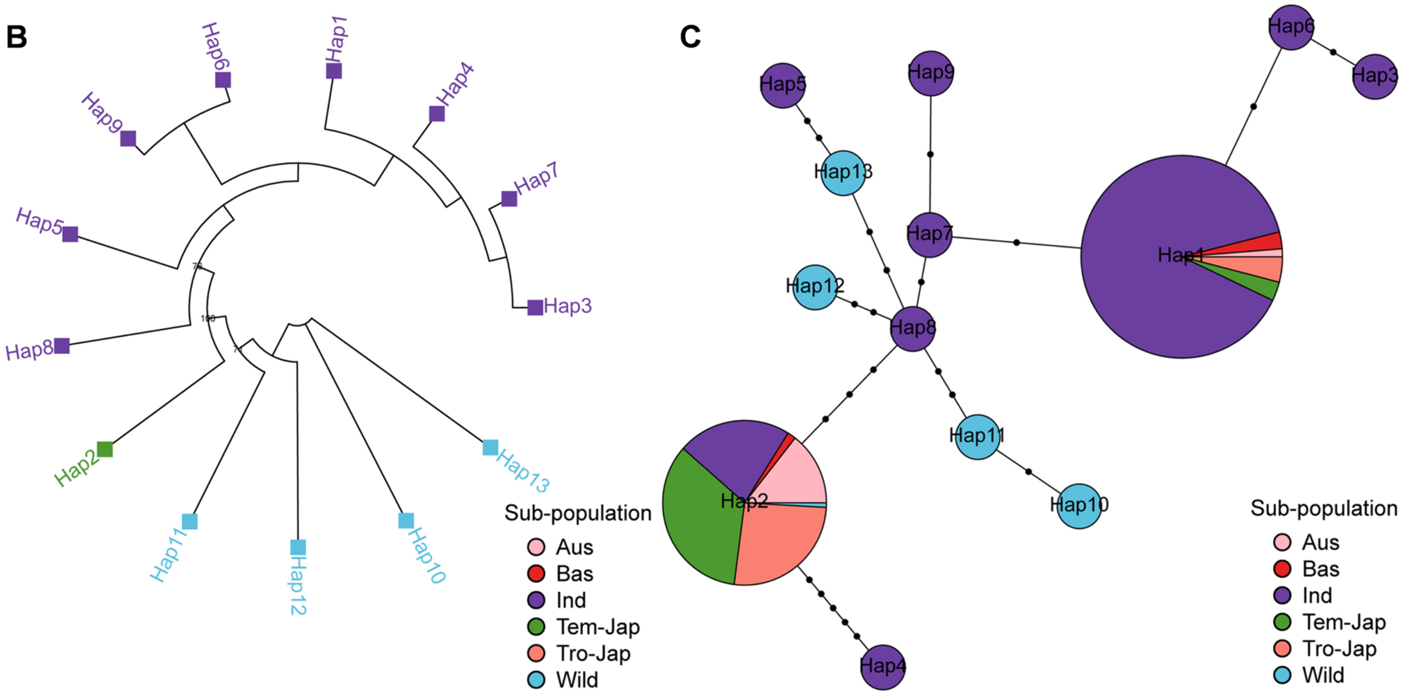

Figure S10. Haplotype analysis of *Fd-GOGAT* in rice. (**A**) Haplotype polymorphism matrix of *Fd-GOGAT* promoter and coding regions from 1990 rice accessions. Yellow-highlighted boxes indicate different nucleotides, and white boxes within letters indicate non-polymorphic nucleotide sites, and white boxes within black dots indicate no sequencing coverage. Ind, indica population. Tem-Jap, temperate japonica population. Tro-Jap, tropical japonica population. Bas, Basmati population. (**B**) Phylogenetic tree of the 13 haplotypes from 1972 cultivated and 18 wild rice accessions. Text on nodes = bootstrap %. Branches: cladogram (equal lengths). (**C**) *Fd-GOGAT* haplotype network. The size of each circle is proportional to the sample count per haplotype. Black dots on the connecting lines denote mutational steps between different haplotypes.

A

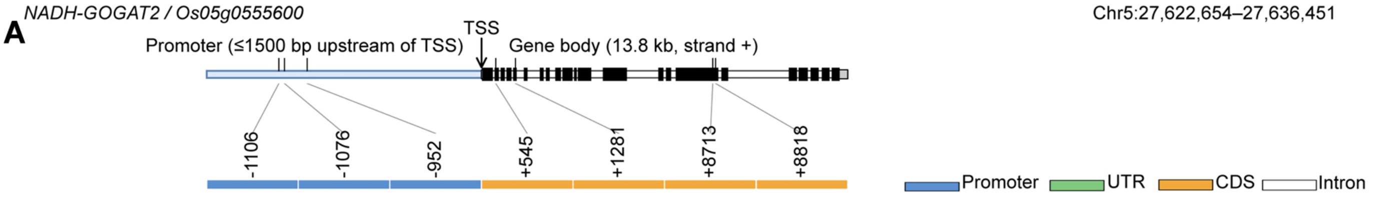

Hap.	n	Promoter (1.5 kb)			Gene body				Sub-population (n)					
		-1106	-1076	-952	+545	+1281	+8713	+8818	Aus	Bas	Ind	Tem-Jap	Tro-Jap	Wild
Hap1	1553	·	·	A	G	T	A	·	127	37	1199	54	136	—
Hap2	298	·	·	G	T	A	A	·	2	4	19	236	37	—
Hap3	100	·	·	A	G	A	A	·	—	5	15	4	76	—
Hap4	5	·	·	·	G	T	A	·	—	—	5	—	—	—
Hap5	5	·	·	A	·	T	A	·	—	—	4	—	1	—
Hap6	5	·	·	A	G	·	A	·	—	—	3	1	1	—
Hap7	2	·	·	A	T	T	A	·	—	—	2	—	—	—
Hap8	2	·	·	·	T	A	A	·	—	—	1	1	—	—
Hap9	2	·	·	G	T	·	A	·	—	—	—	2	—	—
Hap10	1	·	·	G	T	·	·	·	—	—	—	1	—	—
Hap11	1	·	·	A	G	T	·	·	—	—	1	—	—	—
Hap12	1	·	·	G	T	A	·	·	—	—	—	1	—	—
Hap13	1	·	·	·	T	·	A	·	—	—	—	1	—	—
Hap14	1	·	·	·	·	·	A	·	—	—	—	1	—	—
Hap15	1	·	·	A	·	A	A	·	—	—	—	—	1	—
Hap16	1	·	·	·	G	·	A	·	—	—	—	—	1	—
Hap17	1	·	·	G	·	A	A	·	—	—	—	1	—	—
Hap18	3	T	C	·	·	T	G	A	—	—	—	—	—	3
Hap19	2	T	G	·	·	A	A	G	—	—	—	—	—	2
Hap20	2	T	·	·	·	A	A	G	—	—	—	—	—	2
Hap21	2	T	C	·	·	T	A	G	—	—	—	—	—	2
Hap22	2	T	C	·	·	A	A	G	—	—	—	—	—	2
Hap23	2	T	G	·	·	T	A	G	—	—	—	—	—	2
Hap24	1	·	G	·	·	·	A	G	—	—	—	—	—	1
Hap25	1	A	C	·	·	A	A	G	—	—	—	—	—	1
Hap26	1	·	C	·	·	A	A	G	—	—	—	—	—	1
Hap27	1	T	G	·	·	A	G	A	—	—	—	—	—	1

B

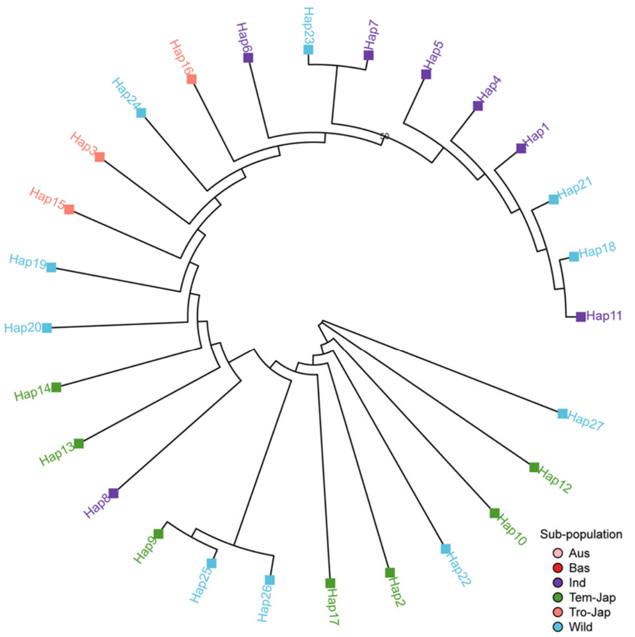

C

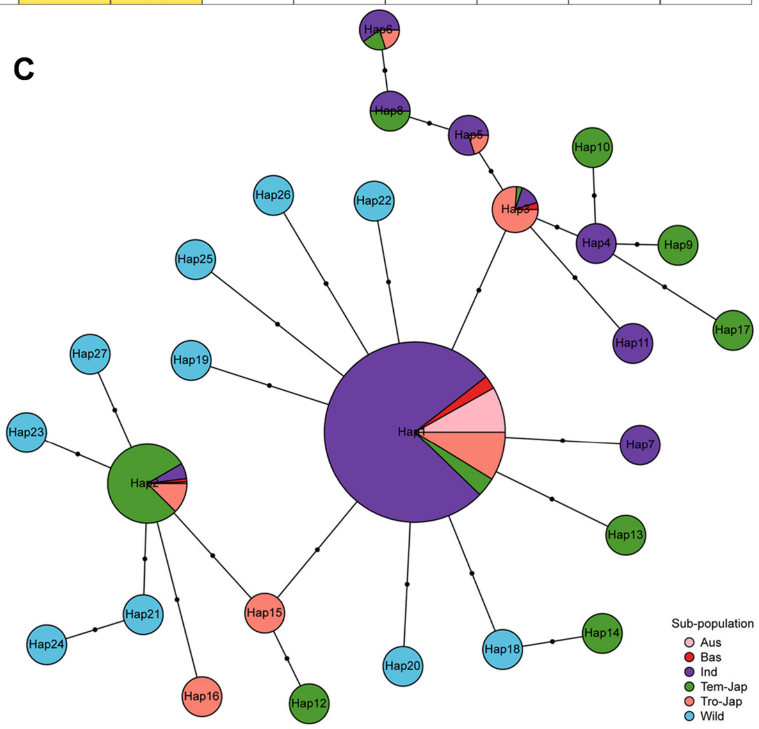

Figure S11. Haplotype analysis of *NADH-GOGAT2* in rice. (**A**) Haplotype polymorphism matrix of *NADH-GOGAT2* promoter and coding regions from 1997 rice accessions. Yellow-highlighted boxes indicate different nucleotides, and white boxes within letters indicate non-polymorphic nucleotide sites, and white boxes within black dots indicate no sequencing coverage. Ind, indica population. Tem-Jap, temperate japonica population. Tro-Jap, tropical japonica population. Bas, Basmati population. (**B**) Phylogenetic tree of the 27 haplotypes from 1980 cultivated and 17 wild rice

accessions. Text on nodes = bootstrap %. Branches: cladogram (equal lengths). (**C**) *NADH-GOGAT2* haplotype network. The size of each circle is proportional to the sample count per haplotype. Black dots on the connecting lines denote mutational steps between different haplotypes.
